# Identification of long noncoding natural antisense transcripts (lncNATs) correlated with drought stress response in wild rice (*Oryza nivara*)

**DOI:** 10.1186/s12864-021-07754-4

**Published:** 2021-06-08

**Authors:** Yong-Chao Xu, Jie Zhang, Dong-Yan Zhang, Ying-Hui Nan, Song Ge, Ya-Long Guo

**Affiliations:** 1 State Key Laboratory of Systematic and Evolutionary Botany, Institute of Botany, Chinese Academy of Sciences, Beijing, 100093 China; 2 University of Chinese Academy of Sciences, Beijing, 100049 China

**Keywords:** Drought stress, Long noncoding RNA, *O. nivara*, Strand-specific RNA-seq, Wild rice

## Abstract

**Background:**

Wild rice, including *Oryza nivara* and *Oryza rufipogon*, which are considered as the ancestors of Asian cultivated rice (*Oryza sativa*), possess high genetic diversity and serve as a crucial resource for breeding novel cultivars of cultivated rice. Although rice domestication related traits, such as seed shattering and plant architecture, have been intensively studied at the phenotypic and genomic levels, further investigation is needed to understand the molecular basis of phenotypic differences between cultivated and wild rice. Drought stress is one of the most severe abiotic stresses affecting rice growth and production. Adaptation to drought stress involves a cascade of genes and regulatory factors that form complex networks. *O. nivara* inhabits swampy areas with a seasonally dry climate, which is an ideal material to discover drought tolerance alleles. Long noncoding natural antisense transcripts (lncNATs), a class of long noncoding RNAs (lncRNAs), regulate the corresponding sense transcripts and play an important role in plant growth and development. However, the contribution of lncNATs to drought stress response in wild rice remains largely unknown.

**Results:**

Here, we conducted strand-specific RNA sequencing (ssRNA-seq) analysis of Nipponbare (*O. sativa*) and two *O. nivara* accessions (BJ89 and BJ278) to determine the role of lncNATs in drought stress response in wild rice. A total of 1246 lncRNAs were identified, including 1091 coding–noncoding NAT pairs, of which 50 were expressed only in Nipponbare, and 77 were expressed only in BJ89 and/or BJ278. Of the 1091 coding–noncoding NAT pairs, 240 were differentially expressed between control and drought stress conditions. Among these 240 NAT pairs, 12 were detected only in Nipponbare, and 187 were detected uniquely in *O. nivara.* Furthermore, 10 of the 240 coding–noncoding NAT pairs were correlated with genes enriched in stress responsive GO terms; among these, nine pairs were uniquely found in *O. nivara*, and one pair was shared between *O. nivara* and Nipponbare.

**Conclusion:**

We identified lncNATs associated with drought stress response in cultivated rice and *O. nivara*. These results will improve our understanding of the function of lncNATs in drought tolerance and accelerate rice breeding.

**Supplementary Information:**

The online version contains supplementary material available at 10.1186/s12864-021-07754-4.

## Background

Rice is one of the most important crops in the world and a major source of food for billions of people. Asian cultivated rice (*Oryza sativa*) was domesticated from wild rice species, including *Oryza rufipogon* and *Oryza nivara*, and experienced a bottleneck effect that severely reduced its genetic diversity [1] and decreased its viability in the natural environment [2]. In contrast to cultivated rice, wild rice species possess higher genetic diversity, which contributes to its greater resistance to biotic and abiotic stresses, and this characteristic of wild rice is crucial for understanding and improving the stress tolerance of cultivated rice.

Drought stress is one of the most severe abiotic stresses affecting crop yield. In the natural environment, plants adapt to drought stress by employing various strategies, such as speeding up their life cycle to avoid drought stress, reducing water loss, and improving water use efficacy [3]. Drought tolerance is a complex trait involving the regulation of a number of physiological and biochemical processes, including stomatal density [4], leaf rolling [5], osmotic adjustment [6], and root system development [7], at different development stages. These mechanisms of drought stress response involve genes belonging to various families including *WRKY*, *MYB*, *NAC*, *ABRE*, *PP2C*, and *SnRK2* [8–13].

Long noncoding RNAs (lncRNAs) constitute a large fraction of the transcriptome that does not encode proteins [14], and play important roles in various biological processes, such as genome stability [15], vernalization [16], telomere maintenance [17], transcriptional activation [18], and other developmental processes [19]. Various transcriptome studies have revealed that pervasive transcription from noncoding transcripts can give rise to functional lncRNAs [20]. lncRNAs are classified as long intergenic noncoding RNAs (lincRNAs) and long noncoding natural antisense transcripts (lncNATs) [21]. lncNATs are transcribed from the opposite strand of sense RNA in the same genomic regions, and may regulate the expression of sense RNA [22–24]. For example, lncNATs affect genes on the opposite strand and increase starch content and grain weight in rice [25]. Furthermore, lncNATs have been studied at the genomic level in many species, including human (*Homo sapiens*) [26], mouse (*Mus musculus*) [27, 28], rice [29–32], *Arabidopsis thaliana* [21, 33], maize (*Zea mays*) [34], *Plasmodium falciparum* [35], and yeast (*Saccharomyces cerevisiae*) [36]. In particular, lncNATs are correlated with the response to various abiotic and biotic stresses [29, 34, 37]. For example, a recent study in Arabidopsis revealed that NATs affect plant thermotolerance [38].

Although our understanding of drought stress response in plants has improved substantially, this topic needs to be investigated further, given the ongoing global climate change. Firstly, more genetic variability that contributes to drought tolerance needs to be identified in the plant germplasm. Secondly, since most of the previous studies focused on cultivated rice, the drought stress response in wild rice remains largely unknown and needs special attention.

Here, we performed strand-specific RNA sequencing (ssRNA-seq) analysis of Nipponbare (*O. sativa* ssp. *japonica*; hereafter referred to as Nip) and two accessions (BJ89 and BJ278) of *O. nivara* collected from its native habitats of Cambodia and Laos. *O. nivara* inhabits swampy areas with a seasonally dry climate [39]. We identified 1091 coding–noncoding NAT pairs, of which 240 pairs were differentially expressed between control and drought stress conditions, and 187 pairs were specifically found in *O. nivara.* Furthermore, according to the GO enrichment analysis of sense transcripts, 10 coding–noncoding NAT pairs were correlated with drought stress, of which nine pairs were uniquely identified in BJ89 and BJ278. Thus, we identified numerous lncNATs correlated with drought stress in *O. nivara*. These lncNATs potentially play important roles in the response to drought stress, and will provide new insights into the mechanism of drought tolerance in *O. nivara*, thus facilitating breeding the cultivated rice varieties.

## Results

### ssRNA-seq and transcript assembly

**Fig. 1:**
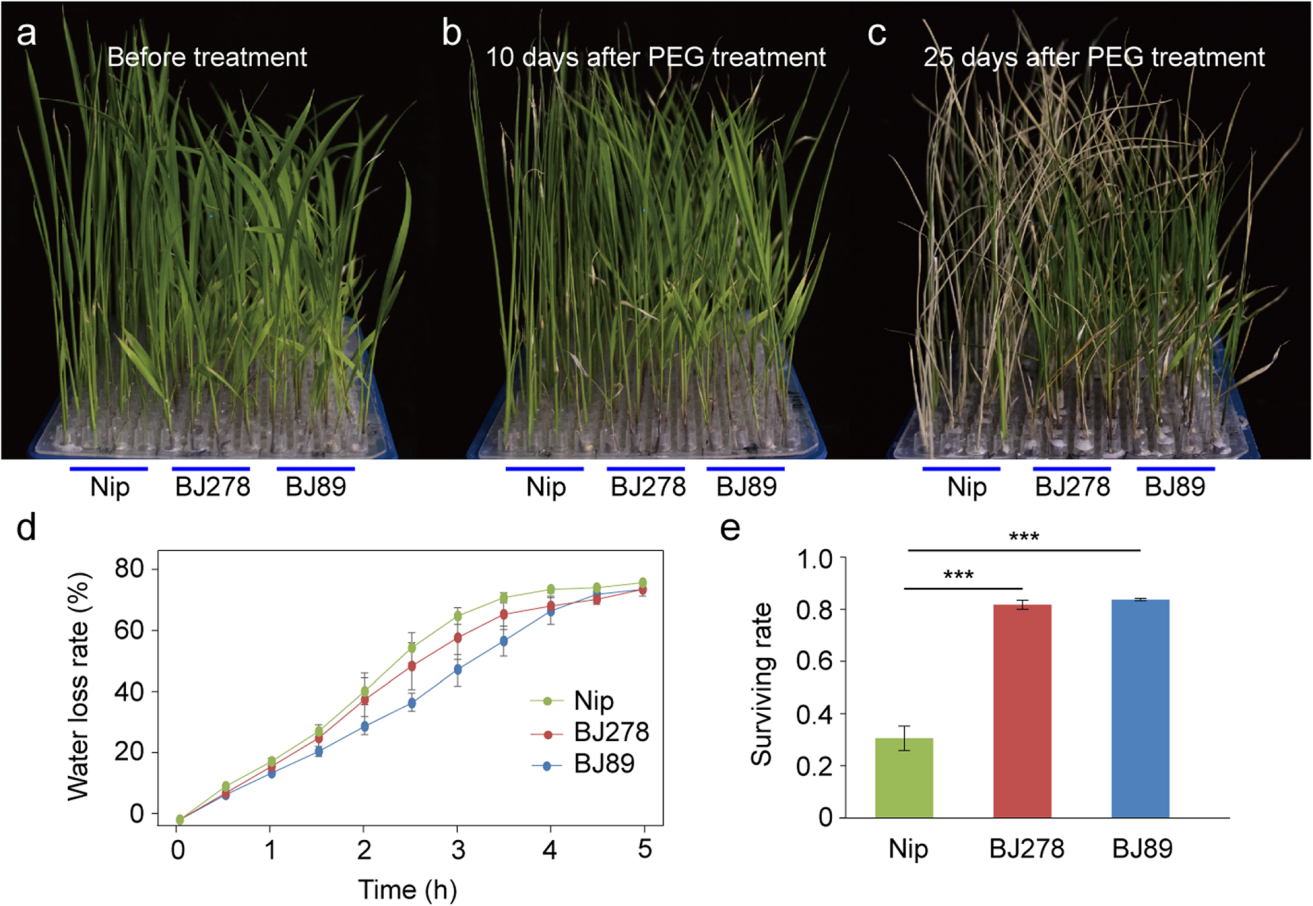
Phenotypic analysis of cultivated rice (Nipponbare; Nip) and *O. nivara* accessions (BJ89 and BJ278) before and after treatment with polyethylene glycol (PEG) to induce drought stress. **a** Photographs of 12-day-old seedlings captured before the PEG treatment. **b**, **c** Photographs of 12-day-old seedlings captured 10 days (**b**) and 25 days (**c**) after the PEG treatment. **d** Water loss rate of leaves. **e** Survival rate of seedlings after the PEG treatment. *** indicates *p*-value < 0.001 performed with Student’s t test

To examine lncNAT expression patterns in wild rice under drought stress treatment, one cultivated rice accession (Nip) and two *O. nivara* accessions (BJ89 and BJ278) were grown under control and drought conditions (Fig. 1a–c). Leaves of BJ89 and BJ278 showed lower water loss than those of Nip (Fig. 1d). Furthermore, the two *O. nivara* accessions exhibited a higher survival rate than Nip after 25 days of drought stress treatment (Fig. 1e). These physiological data suggest that *O. nivara* accessions BJ89 and BJ278 are more drought tolerant than Nip at the seedling stage.

**Fig. 2:**
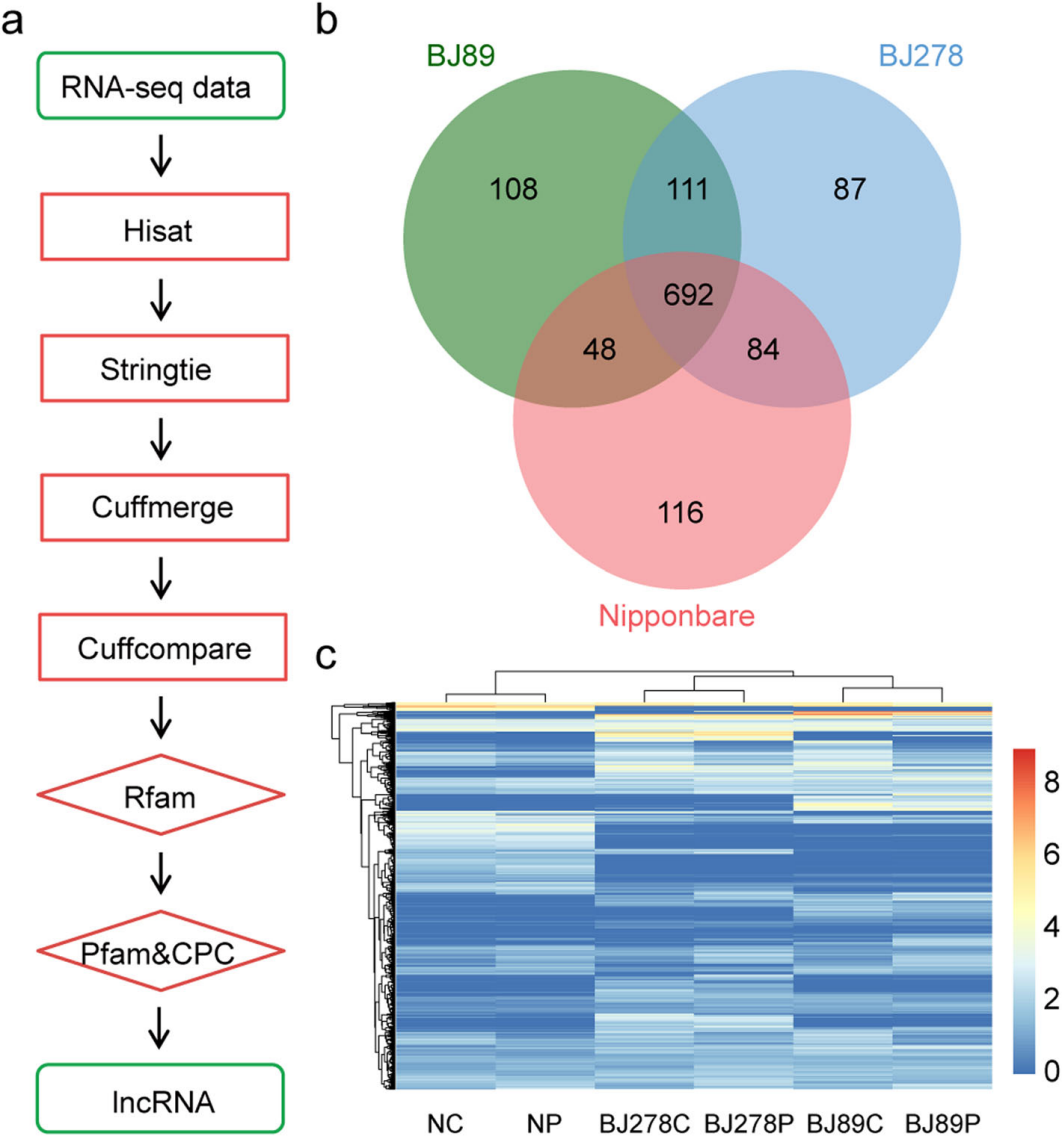
Identification and expression profiles of long noncoding RNAs (lncRNAs) in Nip, BJ89, and BJ278. **a** Flow chart showing the procedure used to identify lncRNAs. **b** Number of lncRNAs in Nip, BJ89, and BJ278. **c** Expression profiles of lncRNAs in Nip, BJ89, and BJ278 under control and drought stress conditions. NC and NP indicate Nip samples under control and drought stress conditions, respectively; BJ278C and BJ278P represent BJ278 samples under control and drought stress conditions, respectively; BJ89C and BJ89P represent BJ89 samples under control and drought stress conditions, respectively

Next, we conducted ssRNA-seq analysis of these three accessions treated with or without 25% polyethylene glycol (PEG-4000; w/v) for 10 days. A total of 18 strand-specific cDNA libraries were constructed from leaf tissues, with three replicates per accession in both control and drought stress treatments. In total, 445.5 million paired-end reads (2 × 125 bp) were generated by ssRNA-seq using Illumina HiSeq 2500, of which 373.6 million reads (83.9%) mapped perfectly on to the Nip reference genome (Table S1). Pearson correlation coefficients of the three biological replicates of each accession were greater than 0.9, indicating the high reproducibility of our ssRNA-seq data (Fig. S1). Among the 373.6 million paired-end reads, we identified 62,201 transcripts, including 17,583 novel transcripts (8905 known gene loci and 2704 new gene loci) with hisat2 [40] and stringtie [41] (Fig. 2a). We also conducted principal component analysis (PCA) of gene expression data. The results showed that PC2 clearly distinguished between the control and drought treated samples, while PC3 separated the different accessions (Fig. S2).

### Identification of lncRNAs

To identify lncRNAs, novel transcripts larger than 200 nt were mapped against the Rfam 13.0 database to exclude micro RNAs (miRNAs), ribosomal RNAs (rRNAs), and other small noncoding RNAs [42]. Then, any transcripts with a coding potential, according to Coding Potential Calculator (CPC) [43] and Pfam with HAMMER scan [44], were filtered out (Fig. 2a). Finally, a total of 1246 lncRNAs were identified, including 940 in Nip, 959 in BJ89, and 974 in BJ278 (reads in the same accession under both control and drought stress conditions were combined to identify lncRNAs) (Fig. 2b and Table S2). Among the 1246 lncRNAs, 692 (55.5%) were common to all three accessions, 306 (24.6%) were uniquely found in at least one of the two *O. nivara* accessions, and 111 (8.9%) were present in both *O. nivara* accessions (Fig. 2b). The expression profiles of lncRNAs were more different between the three accessions than between the drought and control conditions of the same accession (Fig. 2c).

**Fig. 3:**
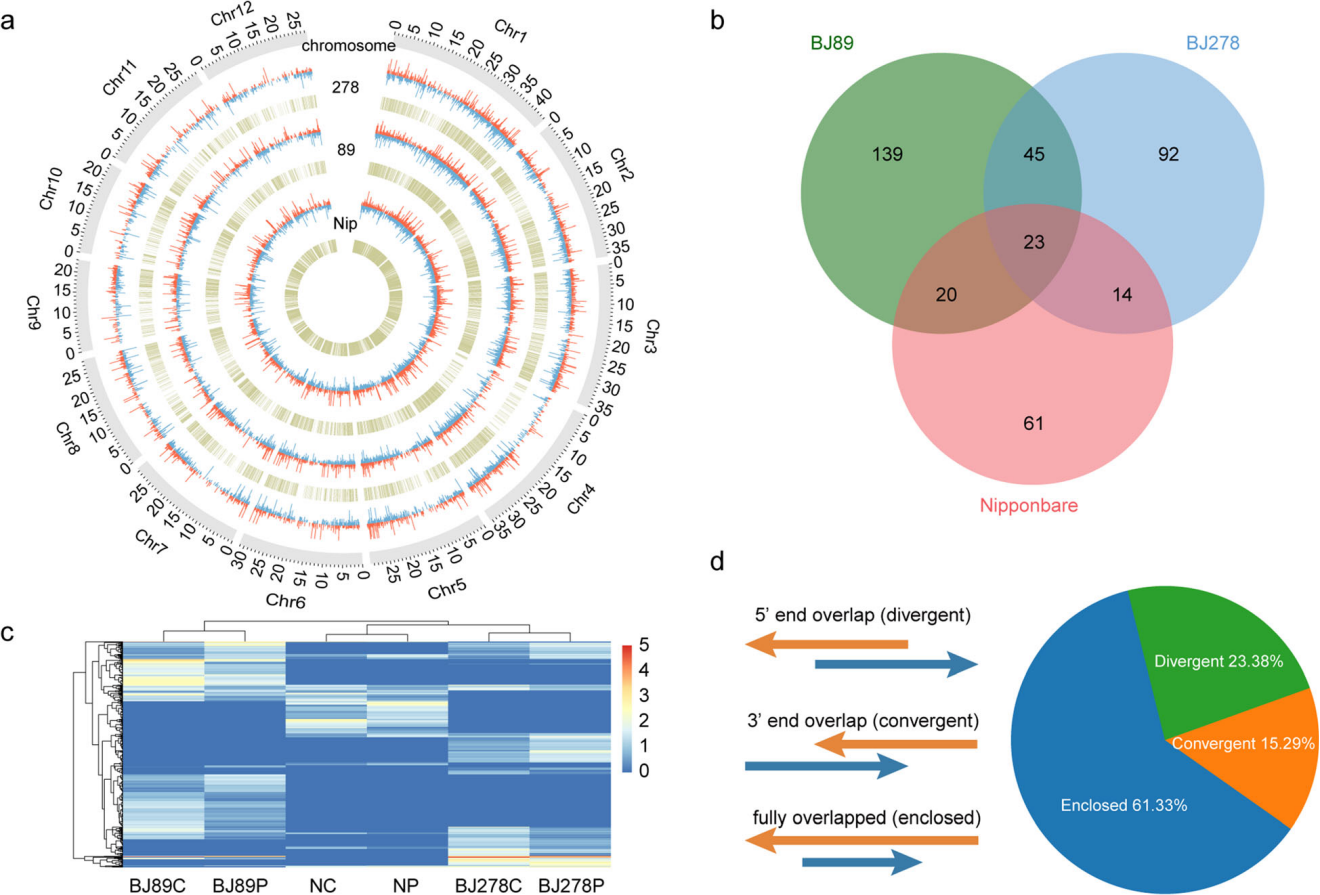
Genome wide identification of natural antisense transcript (NAT) pairs in Nip, BJ89, and BJ278. **a**, **b** Expression patterns. Red and blue lines represent up- and downregulated genes, respectively, under drought stress respectively. Yellow lines indicate the genomic positions of NAT pairs. (**a**) and Venn diagrams (**b**) of differentially expressed lncRNAs in the three accessions. **c** Genome-wide distribution of NAT pairs and differentially expressed genes (DEGs) under drought stress conditions. **d** Classification of NAT pairs according to their direction of transcription and the overlap region between sense and antisense transcripts. Orange arrows indicate sense transcripts, and blue arrows indicate antisense transcripts

Of the 1246 lncRNAs, a total of 394 lncRNAs were differentially expressed between control and drought stress treatments (118 in Nip, 227 in BJ89, and 174 in BJ278). Among these 394 lncRNAs, 23 were common to all three accessions; 34 were identified as being shared between one of the *O. nivara* accessions and Nip; 45 were shared between the two *O. nivara* accessions (BJ89 and BJ278); and 139, 92, and 61 were uniquely found in BJ89, BJ278, and Nip, respectively (Fig. 3a, b).

### Identification of lncNATs and NAT pairs

Based on their location relative to the gene coding regions, 675 of the 1246 lncRNAs were long intergenic noncoding RNAs (lincRNAs; lncRNAs located in intergenic regions), and 571 lncRNAs were long noncoding natural antisense transcripts (lncNATs; lncRNAs overlapped with coding genes on the opposite DNA strand) (Table S2). The lincRNAs contained fewer exons than lncNATs; 72.7% lincRNAs contained only one exon (Fig. S3). By contrast, mRNAs contained more exons than both lincRNAs and lncNATs (Fig. S3). In addition, mRNAs showed higher expression variation than lincRNAs and lncNATs under either control or drought stress condition at the genome level (Fig. S4).

It has been shown that lncNATs can regulate the expression of sense transcripts [45, 46], and each strand of a NAT pair could potentially represent a protein-coding gene. Therefore, in addition to identifying NAT pairs from lncRNAs, we scanned NAT pairs across the whole genome of the three accessions, based on the annotation of the Nip reference genome. All transcripts annotated in the Nip reference genome were integrated and assembled with the ssRNA-seq data generated in this study. A total of 8529 NAT pairs with overlapping regions greater than 25 nt were identified according to a previous study [31]. According to the coding capacity of the sense–antisense pair [27], 86.88% (7410) of NAT pairs were coding–coding pairs (both transcripts with protein-coding capacity), 0.33% (28) were noncoding–noncoding pairs (both transcripts represented lncRNAs), and 12.79% (1091) were coding–noncoding pairs (one strand showed protein-coding capacity, while the other strand represented an lncRNA) (Table S3), and each transcript (transcripts with protein-coding capacity or lncRNAs) could flank a few different transcripts. Depending on the direction and location of the sense and antisense transcripts, 61.33% of the 8529 NAT pairs were enclosed (one transcript fully embedded in the other), 23.38% were divergent (head-to-head, 5′-end overlap), and 15.29% were convergent (tail-to-tail, 3′-end overlap) (Fig. 3d).

Of the 8529 NAT pairs, 5866 were detected through ssRNA-seq analysis of the three accessions, of which 4783, 4813, and 4596 were detected in Nip, BJ89, and BJ278, respectively (Fig. 3c, Fig. S5, Table S3). Of the 5866 NAT pairs, 62.2% (3651) were shared among all three accessions (2792 coding–coding pairs, 13 noncoding–noncoding pairs, and 846 coding–noncoding pairs) (Fig. S5, Table S3); 8.8% (517) were expressed only in Nip (462 coding–coding pairs, 5 noncoding–noncoding pairs, and 50 coding–noncoding pairs) (Fig. S5, Table S3); and 18.5% (1083) were uniquely expressed in *O. nivara* accessions (998 coding–coding pairs, 8 noncoding–noncoding pairs, and 77 coding–noncoding pairs) (Fig. S5, Table S3).

### NAT pairs responsive to drought stress

To clarify the response of NAT pairs to drought stress, we first determined the differences in gene expression patterns between control and drought treatments. In detail, we identified differentially expressed genes (DEGs) using the following criteria: fold change (FC) ≥ 2.0 and false discovery rate (FDR) ≤ 0.01. A total of 3934 (4110 transcripts), 5880 (6235 transcripts), and 5036 (5294 transcripts) DEGs were identified between control and drought treatments in Nip, BJ89, and BJ278, respectively (Fig. 3a, Table S4). To identify genes within biological processes related to drought stress, GO enrichment analysis was performed on all DEGs (FC ≥ 2.0 and FDR ≤ 0.01) and highly differentially expressed genes (HDEGs) (FC ≥ 4.0 and FDR ≤ 0.01) identified in each accession. Based on all DEGs, a total of 57 GO terms in the three accessions, and most terms were related to primary metabolic pathways essential for plant growth and development, such as ‘biosynthetic process’, ‘cellular biosynthetic process’, and ‘primary metabolic process’ (Fig. S6). In addition, different GO terms were enriched in the three accessions in response to drought; for example, the ‘response to water’ GO term was uniquely enriched in BJ278 (Fig. S6).

**Fig. 4:**
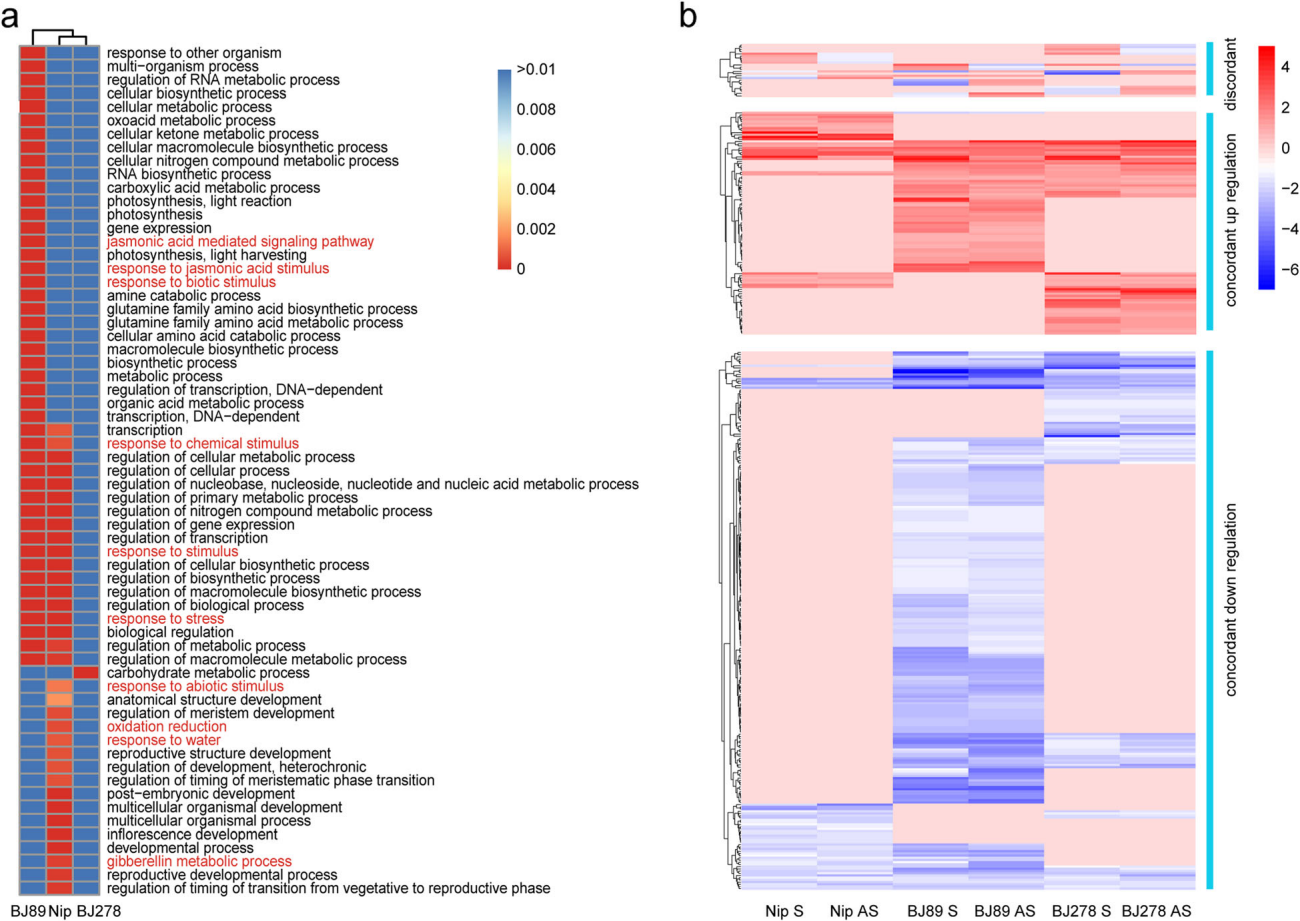
Analysis of drought stress-responsive genes and NAT pairs. **a** Gene ontology (GO) enrichment analysis of DEGs identified in Nip, BJ89, and BJ278 under drought stress conditions. Colors of the heatmap indicate *P*-values of GO enrichment results. GO terms related to drought stress are highlighted in red. **b** Discordant and concordant NAT pairs under drought stress. Pink rectangles indicate NAT pairs showing no difference in expression between drought stress and control conditions. Nip S and Nip AS indicate the expression fold change of sense and antisense transcript between control and drought stress conditaions, respectively. The same as BJ89 S, BJ89 AS, BJ278 S and BJ278 AS

Based on the HDEGs (FC ≥ 4.0 and FDR ≤ 0.01), 63 GO terms in the biological process category were enriched, including 10 terms related to stress, such as ‘response to jasmonic acid stimulus’, ‘oxidation reduction’, and ‘gibberellin metabolic process’ [47, 48] (Fig. 4a, Table S5). Among these ten stress related terms, three (‘response to chemical stimulus’, ‘response to stimulus’, and ‘response to stress’) were detected in both BJ89 and Nip; three terms (‘jasmonic acid mediated signaling pathway’, ‘response to jasmonic acid stimulus’, and ‘response to biotic stimulus’) were uniquely enriched in BJ89; and four terms (‘response to abiotic stimulus’, ‘oxidation reduction’, and ‘response to water and gibberellin metabolic process’) were only enriched in Nip. In BJ278, only one GO term (‘carbohydrate metabolic process’) was enriched. These results suggest that Nip, BJ89, and BJ278 employ different mechanisms to respond to drought stress (Fig. 4a, Fig. S6). A total of 134 HDEGs were enriched in these 10 stress related GO terms. Among these 134 genes, 48 were found only in *O. nivara* (either one or both accessions); 12 were found only in Nip; 35 were shared between Nip and one of the two *O. nivara* accessions (Table S5). In addition, there are 39 genes that are differentially expressed in all three accessions between control and drought treatment. However, these 39 genes exhibit more stronger response in *O. nivara* (Table S5). Antisense transcription could silence or concordantly regulate the sense transcripts [27]. To detect NAT pairs responsive to drought stress, both sense and antisense transcripts of each NAT pair showing differential expression (FC ≥ 2.0 and FDR ≤ 0.01) between control and drought stress conditions were identified as differentially expressed NAT pairs. A total of 369 differentially expressed NAT pairs were identified, of which 240 were coding–noncoding pairs (193 in BJ89, 96 in BJ278, and 53 in Nip). Additionally, among these 240 differentially expressed NAT pairs, 23 were common in all accessions; 18 were shared between Nip and BJ89 or BJ278; 12 were present only in Nip; and 187 were found only in *O. nivara* accessions (Fig. S7, Table S6).

According to the effect of antisense transcripts on sense transcripts, we classified the differentially expressed NAT pairs into two categories, as described previously [21]: discordant (sense and antisense transcripts showing opposite expression patterns) and concordant (sense and antisense transcripts expressed coordinately). A total of 24 discordant NAT pairs were identified (one shared among all accessions; two shared between BJ89 and BJ278; one shared between Nip and BJ278; 6, 6, and 8 uniquely found in Nip, BJ89, and BJ278, respectively) (Fig. S8), including one NAT pair discordant in Nip but concordant in BJ89, and one pair discordant in BJ89 and BJ278 but concordant in Nip (Fig. 4b and Table S6). Additionally, 102 concordant NAT pairs were upregulated (30 in Nip including one downregulated in BJ89, 61 in BJ89, and 54 in BJ278) (Fig. 4b). Among the upregulated concordant NAT pairs, 10 were shared among all accessions, 7 were shared between Nip and BJ278, 13 were found only in Nip, and 72 were found only in *O. nivara* (Fig. S9 and Table S6). A total of 245 concordant NAT pairs were downregulated (45 in Nip, 205 in BJ89, and 82 in BJ278), of which 17 were shared among all three accessions, 14 were shared between Nip and BJ89 or BJ278, 14 were found only in Nip, and 200 were found only in *O. nivara* (Fig. 4b, Fig. S10, and Table S6). RT-qPCR validated that the ssRNA-seq results based on the two samples (Nip and BJ278) for two NAT pairs that could be validated based on RT-qPCR using sequence specific primers (Fig. S11).

Among the 10 coding–noncoding NAT pairs related to GO enrichment terms for the drought stress response, nine were uniquely found in *O. nivara*, and one was common to all accessions (Table 1 Fig. S12, see Fig. S13 for the sequence alignment of the 10 NAT pairs). Furthermore, among the nine coding–noncoding NAT pairs uniquely found in *O. nivara*, six were correlated with response to stress, three were correlated with the jasmonic acid stimulus pathway, and one was correlated with oxidation reduction (Table 1).

**Table 1:** NAT pairs that showing correlation with drought stress response

Chromosome	Sense transcript	Start	End	Antisense transcript	Start	End	GO	Accessions
chr01	MSTRG.1295.1	8,546,015	8,546,972	Os01t0256500–02	8,546,065	8,546,869	GO:0006950 response to stress	BJ89
chr03	MSTRG.12385.1	16,407,687	16,410,061	Os03t0402800–01	16,407,699	16,410,007	GO:0009753 response to jasmonic acid stimulus	BJ89, BJ278
chr03	MSTRG.12385.1	16,407,687	16,410,061	Os03t0402800–02	16,407,695	16,408,985	GO:0009753 response to jasmonic acid stimulus	BJ89
chr03	Os03t0161900–01	3,342,277	3,344,547	MSTRG.10570.1	3,342,143	3,344,510	GO:0006950 response to stress	BJ89
chr03	Os03t0161900–02	3,342,254	3,344,542	MSTRG.10570.1	3,342,143	3,344,510	GO:0006950 response to stress	BJ89
chr04	Os04t0497700–01	24,889,983	24,891,470	MSTRG.16704.1	24,889,504	24,891,481	GO:0055114 oxidation reduction	BJ89
chr07	Os07t0615200–01	25,348,060	25,350,242	MSTRG.27311.1	25,348,061	25,350,279	GO:0009867 jasmonic acid mediated signaling pathway	BJ89
chr08	Os08t0504700–01	24,953,937	24,954,919	MSTRG.30183.1	24,953,534	24,955,156	GO:0006950 response to stress	Nip, BJ89, BJ278
chr09	MSTRG.30767.1	656,756	658,373	Os09t0106700–01	656,788	658,373	GO:0006950 response to stress	BJ89
chr09	MSTRG.31075.1	5,538,425	5,540,535	Os09t0273600–00	5,538,561	5,539,063	GO:0006950 response to stress	BJ89

## Discussion

Antisense transcripts are present in various organisms and play important roles in regulating gene expression. For example, the gene encoding the famous transcriptional repressor FLOWERING LOCUS C (FLC), which delays flowering time in Arabidopsis, is repressed at warm temperatures by *COLD INDUCED LONG ANTISENSE INTRAGENIC RNA (COOLAIR)*, an antisense RNA, via histone demethylation [46, 49, 50]. Antisense lncRNAs can also upregulate gene expression; for example, an lncRNA transcribed from promoter region of the *Pcdhα* gene leads to DNA demethylation of the CTCF binding sites and the activation of sense promoters [51]. Natural cis-antisense transcripts also define the function of short interfering RNAs (siRNAs) and affect their biogenesis; for example *P5CDH* and *SRO5* regulate salt tolerance by generating two types of siRNAs in Arabidopsis [37]. In addition, NATs also contribute to heterochromatin formation and DNA methylation, and suppress gene expression in tumorous cells [52]. However, the function of conserved lncRNAs can vary across different species. For example, in human and mouse, the conserved lncRNAs exhibit different subcellular localization patterns [53].

*O. nivara*, one of the wild progenitor species of cultivated rice, inhabits swampy areas with a seasonally dry climate. *O. nivara* possesses greater genetic diversity than cultivated rice and thus is a valuable resource for breeding rice cultivars with desirable traits, such as stress resistance. In this study, we identified 240 coding–noncoding differentially expressed NAT pairs, of which both of the coding genes on one strand and the lncRNAs on the opposite strand were differentially expressed between control and drought stress conditions. Furthermore, we identified 187 coding–noncoding NAT pairs in the leaves of *O. nivara* accessions, of which 10 were correlated with drought stress. These findings are expected to facilitate further analysis of drought stress tolerance in wild rice. However, to identify more drought resistance genes and NAT pairs, a more comprehensive analysis of different tissues and developmental stages of wild rice should be conducted. It is interesting that even the two accessions from *O. nivara* have different NAT pairs. The variation could be resulted from the genetic variation of within this species, which has a much high genetic variation than cultivated *O. sativa*.

Furthermore, these NAT pairs need to be validated through functional analysis, and the functions of a vast majority of NAT pairs remain unknown. Therefore, further studies are needed to understand the molecular mechanism of action of these coding–noncoding NAT pairs and their regulatory network under drought stress conditions.

## Conclusions

In this study, we performed ssRNA-seq analysis of cultivated rice Nipponbare and two *O. nivara* accessions, and systematically identified numerous genes and NAT pairs responsive to drought stress in all three accessions. Overall, the results of this study will enhance our understanding of the evolution of NATs and drought tolerance in rice, thus facilitating rice breeding.

## Methods

### Plant material and growth conditions

*Oryza nivara* accessions BJ89 and BJ278 were collected from Cambodia and Laos, respectively, and were deposited at the PE Herbarium, Institute of Botany, Chinese Academy of Sciences. All experiments were conducted in an environmentally-controlled growth chamber (Percival Scientific, Inc.). Seeds were incubated at 42 °C for at least 5 days to break dormancy. Seeds were then placed on a wet filter paper in a culture dish at 30 °C for 3 days. The most uniformly geminated seeds were sown in a bottom-less 96-well plate placed in a dish containing Kimura B culture solution [54], and incubated in the growth chamber at 30 °C day/25 °C night temperature under a 12-h light/12-h dark photoperiod. The culture solution was renewed every 3 days. To conduct drought stress and control treatments, 12-day-old seedlings were transferred to a Kimura B culture solution containing 25% PEG-4000 (w/v) or no PEG-4000, respectively, and incubated for 10 days. Three biological replications were performed for each accession in each treatment.

### Strand-specific cDNA library preparation and ssRNA-seq

Total RNA was extracted from leaves of five plants that had been subjected to 10-day-drought or control treatments using Promega Total RNA Isolation System (Z3100). Then, mRNA was fragmented in the fragmentation buffer (Ambion). The first strand of cDNA was reversed transcribed with SuperScript II (Invitrogen) using random primer (TAKARA). Next, dTTP was replaced by dUTP for the synthesis of the second strand. The repaired double-stranded cDNA was ligated with Illumina TruSeq adaptor and digested using USER enzyme (NEB). Finally, 350–450 bp fragments were recovered and purified, and 18 cDNA libraries (3 accessions × 2 treatments × 3 replicates) were sequenced on the Illumina HiSeq 2500 platform.

### Measurement of water loss and survival rate

A water loss experiment was conducted to investigate the variation in the rate of water loss rate from leaves among Nip, BJ89 and BJ278. Five topmost fully expanded leaves were collected from 25-day-old seedlings of each accession and weighted every 30 min at room temperature. Three replications were performed for each accession. The relative water loss rate was calculated using the following equation:

$$ Relative\kern0.5em water\kern0.5em loss\kern0.5em rate\kern0.5em =\left(\mathrm{FW}\hbox{-} \mathrm{CW}\right)/\mathrm{FW} $$

where FW and CW represent the fresh and current weight of leaves, respectively.

To examine the differences in survival rate among the three accessions, 12-day-old seedlings of each accession were treated with Kimura B culture solution containing 25% PEG-4000 (w/v) for 4 weeks and then transferred to Kimura B culture solution without PEG for 1 week. The survival rate of each accession was calculated as the ratio of alive plants to all plants.

### Transcript assembly and lncRNA identification

Paired-end (2 × 125 bp) strand-specific reads of each accession were combined and mapped to the Nip reference genome sequence (IRGSP-1.0) using the hisat2 (2.1.0) software [40]. Stringtie was used to assemble transcripts for regions with read coverage greater than 5X [41]. Then, transcripts of the three accessions were integrated using Cuffmerge and Cuffcompare utilities in the Cufflinks package [55]. Gene annotations of the Nip reference genome (http://rapdb.dna.affrc.go.jp) were integrated, and gene expression levels (estimated as FPKM [Fragments Per Kilobase of transcript per Million mapped reads] values) and DEGs were determined using Cuffdiff in the Cufflinks package [56]. Transcripts longer than 200 nt were mapped against the Rfam 13.0 database to exclude miRNAs, rRNAs, and other small noncoding RNAs [42]. Then, Coding Potential Calculator (CPC) [43] and Pfam [44] were used to filter out potential coding transcripts. Gene function annotations were available from the RAP-DB (http://rapdb.dna.affrc.go.jp/index.html). GO enrichment analysis was conducted using agriGO [57].

### Expression analysis

RNA was extracted from 14-day-old seedlings using the SV Total RNA Isolation System (Promega). First-strand cDNA was obtained using RevertAid First Strand cDNA Synthesis Kit (ThermoFisher). RT-qPCR (one cycle of 95 °C for 15 s, followed by 45 cycles of 95 °C for 15 s, 56 °C for 45 s) was performed using the qTOWER^3^ Real-Time PCR Thermal Cycler (Analytik Jena, Germany) with TB Green Premix Ex TaqII (TaKaRa, Japan) according to the manufacturers’ instructions. The 2-ΔΔCT method was used to determine the gene expression level. The expression level of all transcript was normalized against *actin*. Each experiment was repeated with three independent biological replicates, and RT-qPCR reactions were performed with three biological replicates and three technical replicates for each sample. The primers used in this experiment are listed in Table S7.

### Statistical analysis

All statistical analyses were performed using R (http://www.r-project.org/).

## Supplementary Information

**Additional file 1: Fig. S1.** Pearson correlation coefficient of 18 strand-specific RNA-seq (ssRNA-seq) datasets. Gene expression levels (estimated as FPKM [Fragments Per Kilobase of transcript per Million mapped reads] values) were used for this analysis. Three replicates were performed for each accession. NC and NP indicate Nipponbare (cultivated rice; Nip) samples under control and drought stress conditions, respectively; BJ278C and BJ278P represent BJ278 (*O. nivara*; wild rice) samples under control and drought stress conditions, respectively; BJ89C and BJ89P represent BJ89 (*O. nivara*; wild rice) samples under control and drought stress conditions, respectively. **Fig. S2.** Principal component analysis (PCA) of FPKM data of Nip, BJ89, and BJ278 obtained under drought stress and control treatments. NC and NP indicate Nip samples under control and drought stress conditions, respectively; BJ278C and BJ278P represent BJ278 samples under control and drought stress conditions, respectively; BJ89C and BJ89P represent BJ89 samples under control and drought stress conditions, respectively. **Fig. S3.** Number of exons in long intergenic noncoding RNAs (lincRNAs), long noncoding NATs (lncNATs), and mRNAs. **Fig. S4.** Expression levels (log_2_FPKM) of different RNAs in Nip, BJ89, and BJ278. NC and NP indicate Nip samples under control and drought stress conditions, respectively; BJ278C and BJ278P represent BJ278 samples under control and drought stress conditions, respectively; BJ89C and BJ89P represent BJ89 samples under control and drought stress conditions, respectively. Red triangles represent coefficient of variation. **Fig. S5.** Venn diagram of NAT pairs expressed in Nip, BJ89 and BJ278. **Fig. S6.** Gene Ontology (GO) enrichment analysis based on differentially expressed genes (DEGs) under drought stress. Most GO terms were related to primary metabolic pathways. Colors indicate *P*-values of GO terms. **Fig. S7.** Venn diagram of differentially expressed NAT pairs under drought stress in Nip, BJ89 and BJ278. **Fig. S8.** Venn diagram of discordant NAT pairs under drought stress in Nip, BJ89, and BJ278. **Fig. S9.** Venn diagram of upregulated concordant NAT pairs under drought stress in Nip, BJ89, and BJ278. **Fig. S10.** Venn diagram of downregulated concordant NAT pairs under drought stress in Nip, BJ89, and BJ278. **Fig. S11.** The expression level of randomly selected two concordant NAT pairs that could design strand-specific primers. The relative expression level of Os02t0258800–01 (a, sense transcript) and MSTRG.6860.1 (b, antisense transcript) were both increased after PEG treatment. The relative expression level of BJ278 were increased in both Os02t0504000–01(c, sense transcript) and MSTRG.7513.1 (d, antisense transcript) after treatment but the transcripts in Nip showed no difference between control and treatment. “***” indicated that the *p*-value was less than 0.001. **Fig. S12.** Venn diagram of coding–noncoding NAT pairs of which enriched in GO terms related to drought stress in Nip, BJ89, and BJ278. **Fig. S13.** Sequence alignments of 10 coding–noncoding NAT pairs related to GO enrichment terms for the drought stress response. The top sequence was sense transcript and the sequence below was antisense transcript. **Table S1.** Statistics of paired-end reads generated by ssRNA-seq analysis of 18 cDNA libraries of Nipponbare (Nip), BJ89, and BJ278 samples treated with or without drought stress. **Table S7.** List of primers used in this study for quantitative PCR.

**Additional file 2: Table S2.** Different types of long noncoding RNAs (lncRNAs).

**Additional file 3: Table S3.** Natural antisense transcript (NAT) pairs expressed in Nip, BJ89, and BJ278.

**Additional file 4: Table S4.** Differentially expressed genes (DEGs) identified in Nip, BJ89, and BJ278 under the drought stress relative to control conditions.

**Additional file 5: Table S5.** Genes included in 10 Gene Ontology (GO) categories closely related to drought stress. Red color indicates 48 genes differentially expressed only in BJ89 and BJ278 between drought stress and control treatments. Blue color indicates 49 genes differentially expressed between BJ89 and Nip or between BJ278 and Nip under drought stress treatments.

**Additional file 6: Table S6.** NAT pairs differentially expressed in Nip, BJ89, and BJ278 between drought stress and control treatments.

## Data Availability

The ssRNA-seq datasets generated during the current study are available in the China National Center for Bioinformation (https://bigd.big.ac.cn/databases) under the accession number: CRA003736. The samples used in this study were deposited at the PE Herbarium, Institute of Botany, Chinese Academy of Sciences.
