# Supplementary material for: Identification of long noncoding natural antisense transcripts (lncNATs) correlated with drought stress response in wild rice (Oryza nivara)
Source: BMC Genomics. 2021 Jun 8;22:424. doi: 10.1186/s12864-021-07754-4 (PMC8188688; doi:10.1186/s12864-021-07754-4)
Supplement: Supplementary file 1 — Additional file 1: Fig. S1. Pearson correlation coefficient of 18 strand-specific RNA-seq (ssRNA-seq) datasets. Gene expression levels (estimated as FPKM [Fragments Per Kilobase of transcript per Million mapped reads] values) were used for this analysis. Three replicates were performed for each accession. NC and NP indicate Nipponbare (cultivated rice; Nip) samples under control and drought stress conditions, respectively; BJ278C and BJ278P represent BJ278 (O. nivara; wild rice) samples under control and drought stress conditions, respectively; BJ89C and BJ89P represent BJ89 (O. nivara; wild rice) samples under control and drought stress conditions, respectively. Fig. S2. Principal component analysis (PCA) of FPKM data of Nip, BJ89, and BJ278 obtained under drought stress and control treatments. NC and NP indicate Nip samples under control and drought stress conditions, respectively; BJ278C and BJ278P represent BJ278 samples under control and drought stress conditions, respectively; BJ89C and BJ89P represent BJ89 samples under control and drought stress conditions, respectively. Fig. S3. Number of exons in long intergenic noncoding RNAs (lincRNAs), long noncoding NATs (lncNATs), and mRNAs. Fig. S4. Expression levels (log2FPKM) of different RNAs in Nip, BJ89, and BJ278. NC and NP indicate Nip samples under control and drought stress conditions, respectively; BJ278C and BJ278P represent BJ278 samples under control and drought stress conditions, respectively; BJ89C and BJ89P represent BJ89 samples under control and drought stress conditions, respectively. Red triangles represent coefficient of variation. Fig. S5. Venn diagram of NAT pairs expressed in Nip, BJ89 and BJ278. Fig. S6. Gene Ontology (GO) enrichment analysis based on differentially expressed genes (DEGs) under drought stress. Most GO terms were related to primary metabolic pathways. Colors indicate P-values of GO terms. Fig. S7. Venn diagram of differentially expressed NAT pairs under drought stress in Nip, [file 12864_2021_7754_MOESM1_ESM.docx]

Supplementary information for

**Identification of long noncoding natural antisense transcripts (lncNATs) correlated with drought stress response in wild rice (*Oryza nivara*)**

Yong-Chao Xu^1,2,†^, Jie Zhang^1,2,†^, Dong-Yan Zhang^1,2^, Ying-hui Nan^1,2^, Song Ge^1,2^,Ya-Long Guo^1,2*^

^1^ State Key Laboratory of Systematic and Evolutionary Botany, Institute of Botany, Chinese Academy of Sciences, Beijing 100093, China

^2^ University of Chinese Academy of Sciences, Beijing 100049, China

† These author contribute equally to this work

* Corresponding author: Ya-Long Guo

State Key Laboratory of Systematic and Evolutionary Botany

Institute of Botany, Chinese Academy of Sciences

Beijing 100093, China

PH +86-62836298; FX +86-62590843

EM yalong.guo@ibcas.ac.cn

Key words: Drought stress, Long non-coding RNA, *O. nivara*, Strand specific RNA-seq, Wild rice.

Figure S1. Pearson correlation coefficient of 18 strand-specific RNA-seq (ssRNA-seq) datasets. Gene expression levels (estimated as FPKM [Fragments Per Kilobase of transcript per Million mapped reads] values) were used for this analysis. Three replicates were performed for each accession. NC and NP indicate Nipponbare (cultivated rice; Nip) samples under control and drought stress conditions, respectively; BJ278C and BJ278P represent BJ278 (*O. nivara*; wild rice) samples under control and drought stress conditions, respectively; BJ89C and BJ89P represent BJ89 (*O. nivara*; wild rice) samples under control and drought stress conditions, respectively.


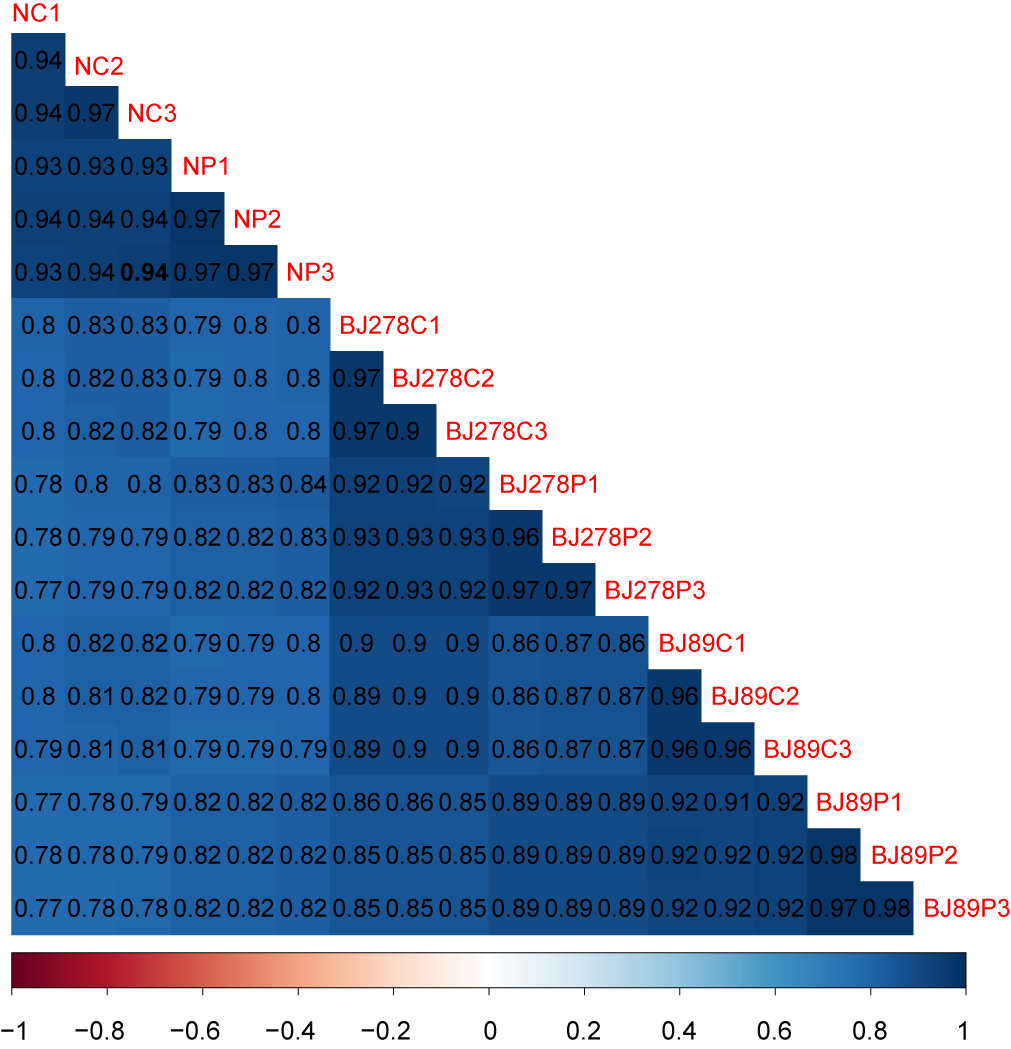


Figure S2. Principal component analysis (PCA) of FPKM data of Nip, BJ89, and BJ278 obtained under drought stress and control treatments. NC and NP indicate Nip samples under control and drought stress conditions, respectively; BJ278C and BJ278P represent BJ278 samples under control and drought stress conditions, respectively; BJ89C and BJ89P represent BJ89 samples under control and drought stress conditions, respectively.


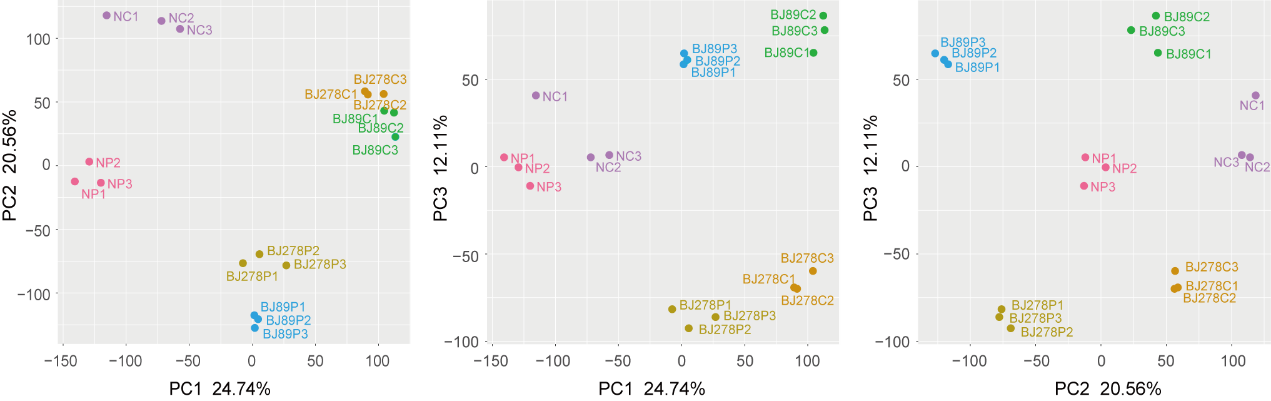


Figure S3. Number of exons in long intergenic noncoding RNAs (lincRNAs), long noncoding NATs (lncNATs), and mRNAs.


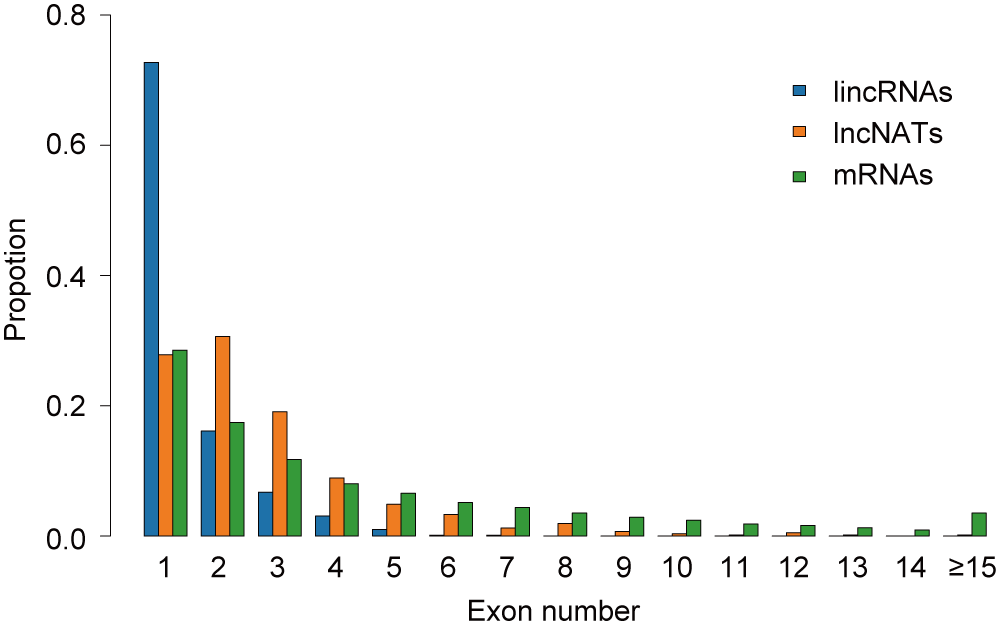


Figure S4. Expression levels (log_2_FPKM) of different RNAs in Nip, BJ89, and BJ278. NC and NP indicate Nip samples under control and drought stress conditions, respectively; BJ278C and BJ278P represent BJ278 samples under control and drought stress conditions, respectively; BJ89C and BJ89P represent BJ89 samples under control and drought stress conditions, respectively. Red triangles represent coefficient of variation.


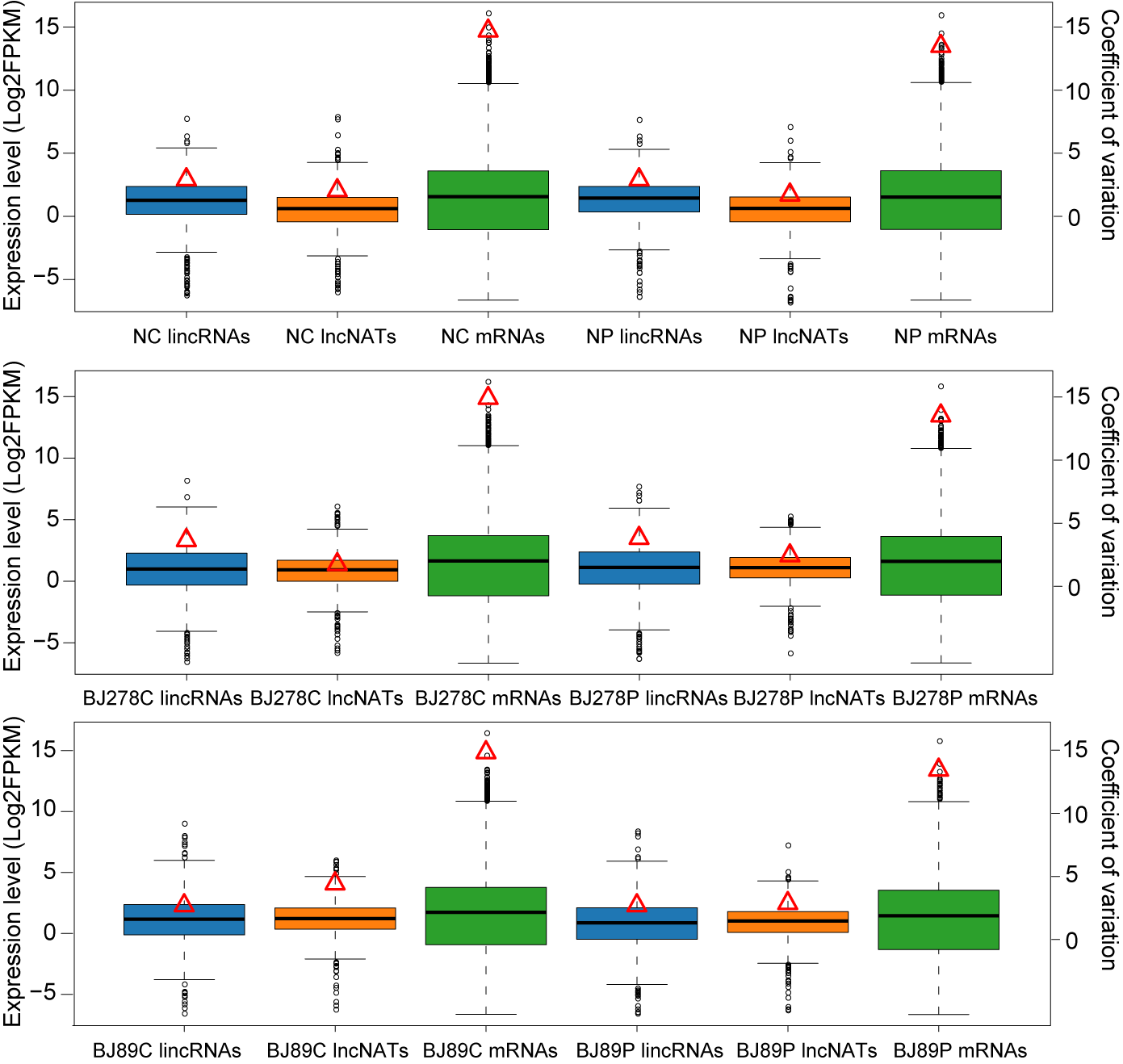


Figure S5. Venn diagram of NAT pairs expressed in Nip, BJ89 and BJ278.


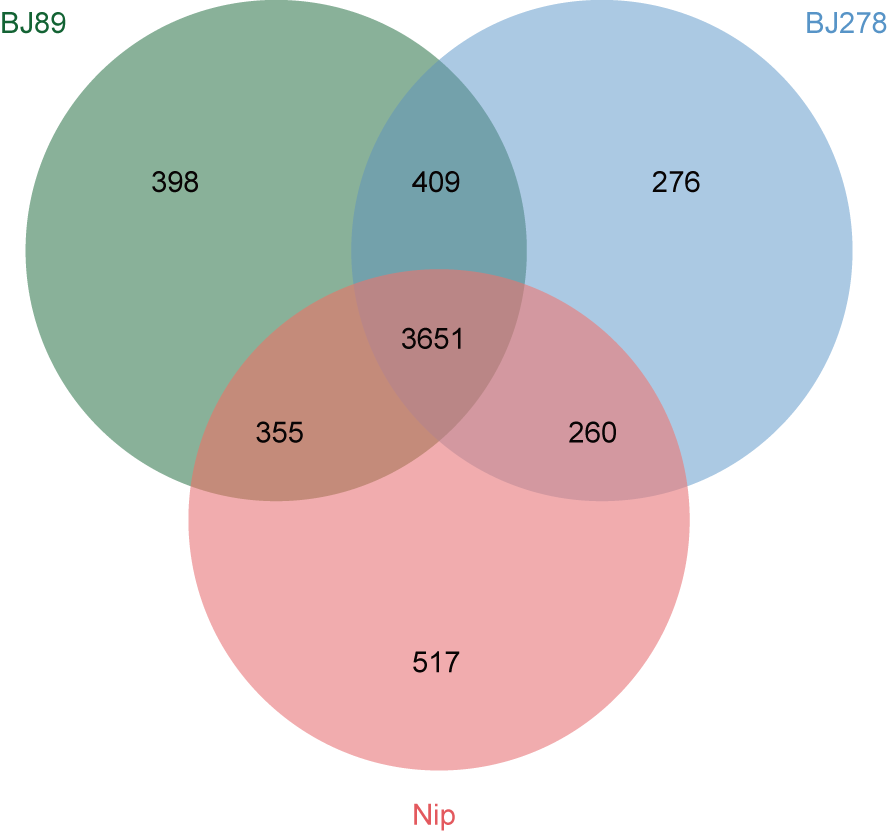


Figure S6. Gene Ontology (GO) enrichment analysis based on differentially expressed genes (DEGs) under drought stress. Most GO terms were related to primary metabolic pathways. Colors indicate *P*-values of GO terms.


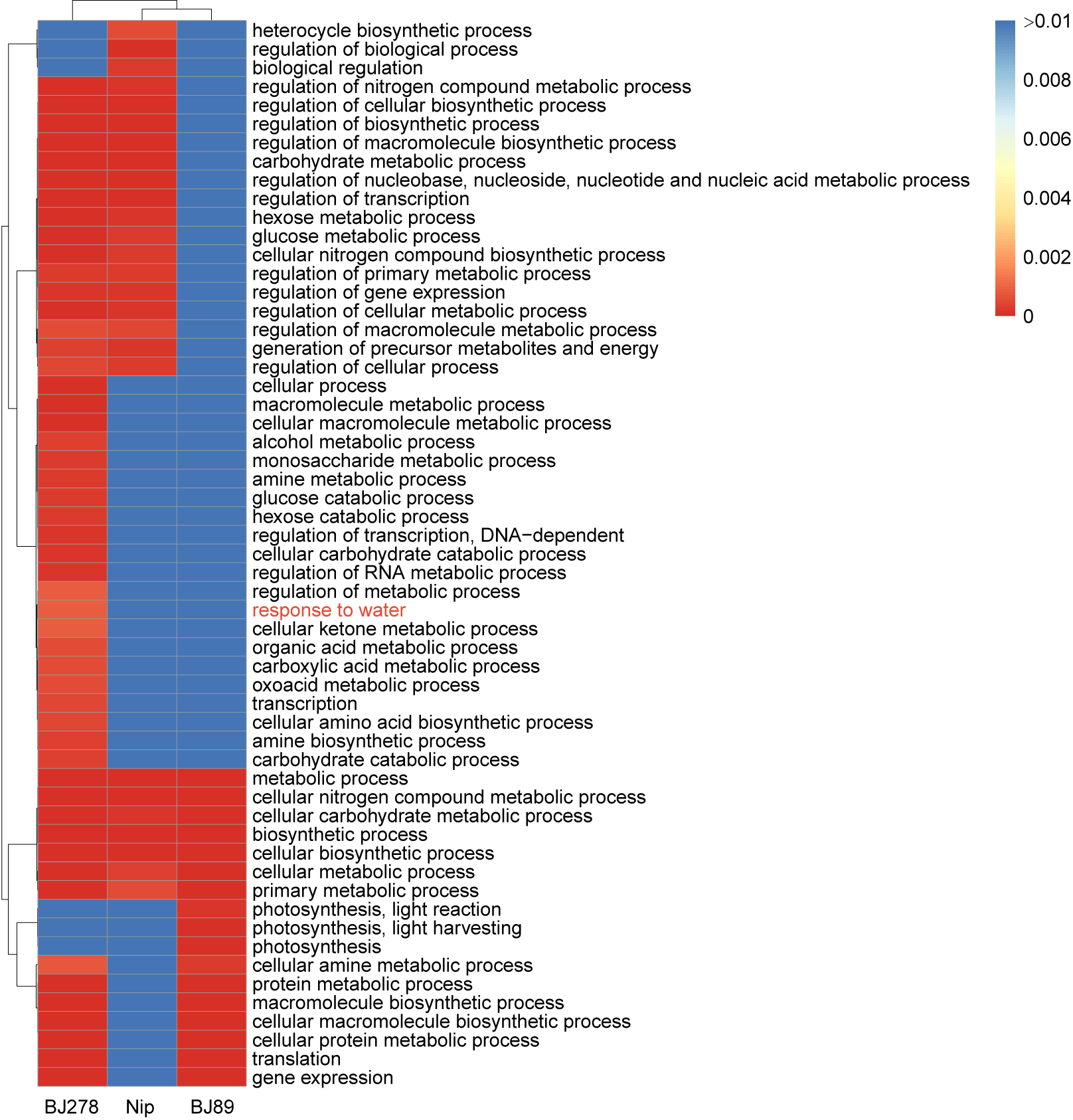


Figure S7. Venn diagram of differentially expressed NAT pairs under drought stress in Nip, BJ89 and BJ278.


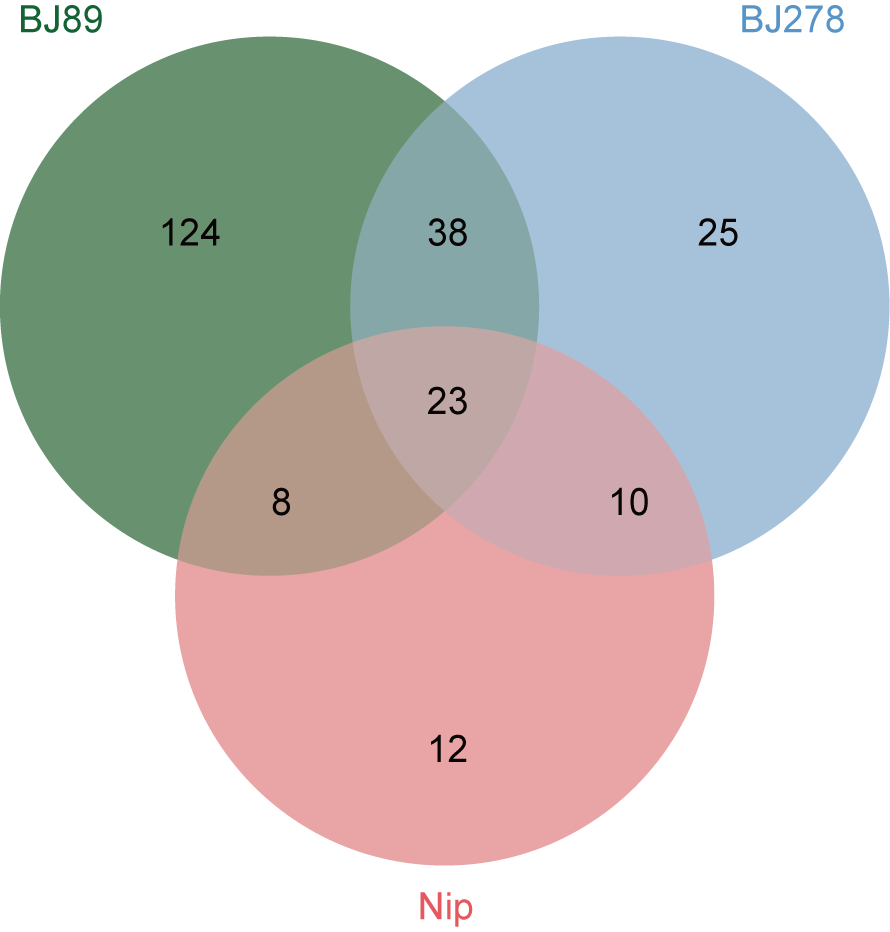


Figure S8. Venn diagram of discordant NAT pairs under drought stress in Nip, BJ89, and BJ278.


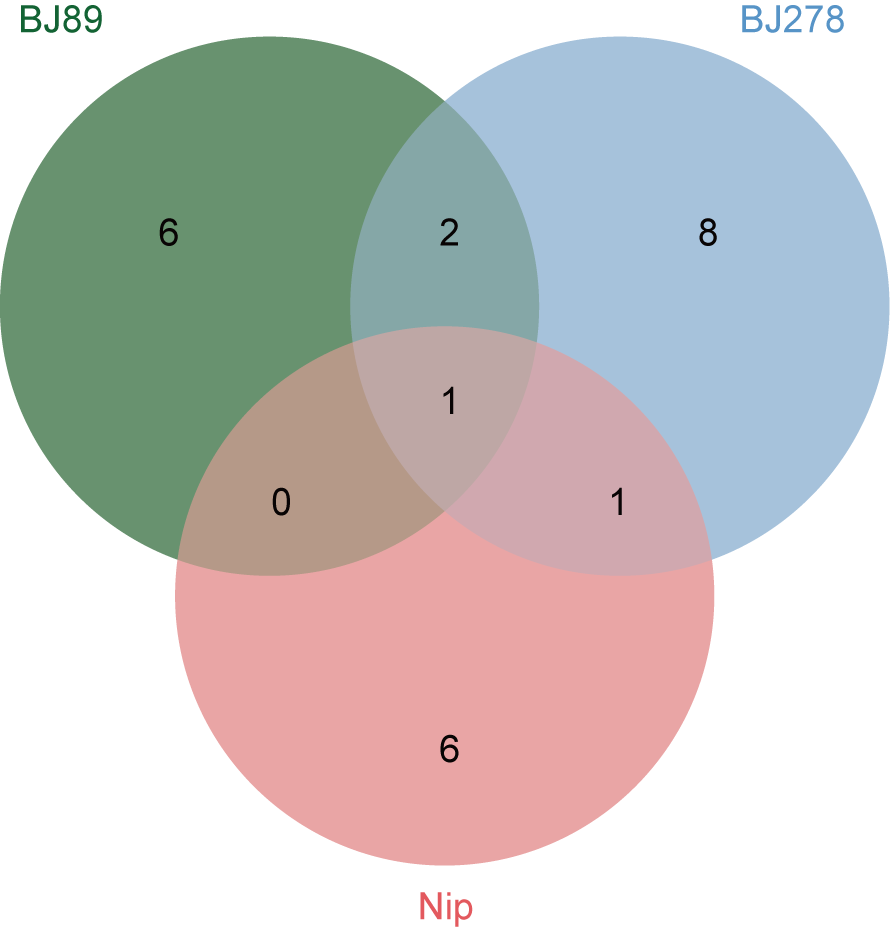


Figure S9. Venn diagram of upregulated concordant NAT pairs under drought stress in Nip, BJ89, and BJ278.


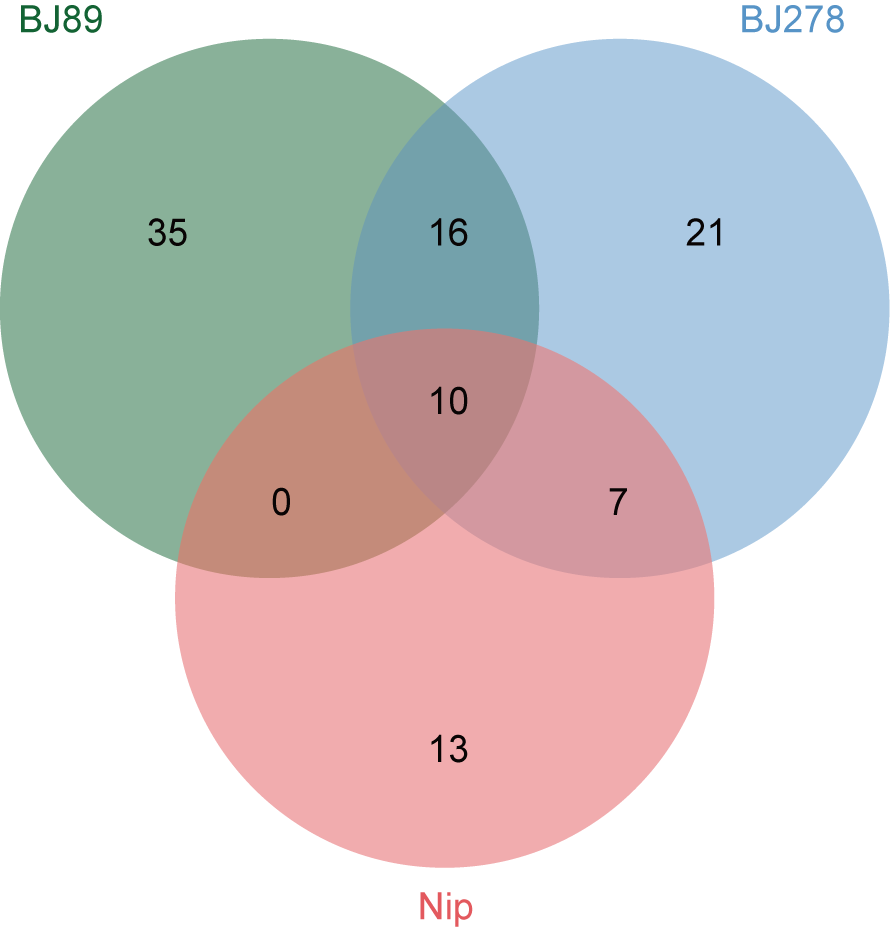


Figure S10. Venn diagram of downregulated concordant NAT pairs under drought stress in Nip, BJ89, and BJ278.


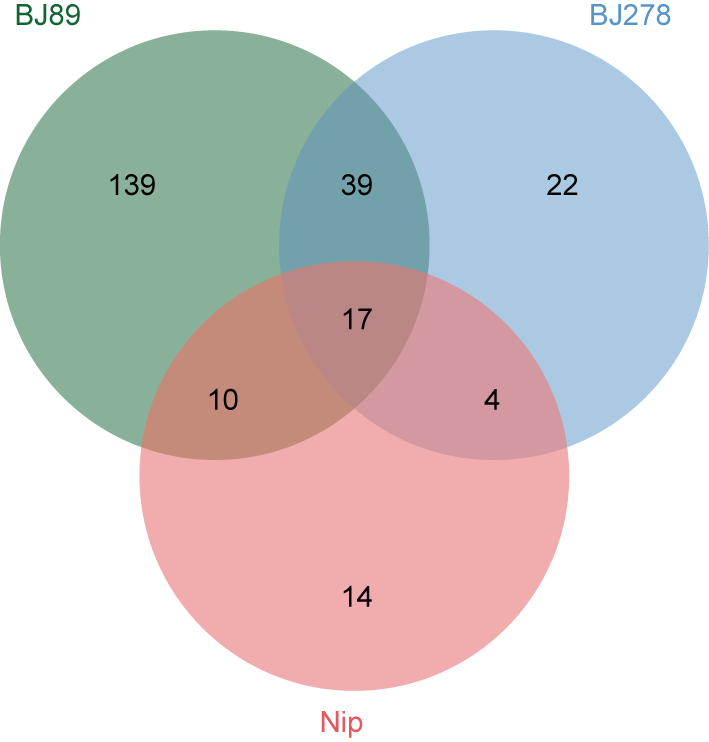


Figure S11. The expression level of randomly selected two concordant NAT pairs that could design strand-specific primers. The relative expression level of Os02t0258800-01 (a, sense transcript) and MSTRG.6860.1 (b, antisense transcript) were both increased after PEG treatment. The relative expression level of BJ278 were increased in both Os02t0504000-01(c, sense transcript) and MSTRG.7513.1 (d, antisense transcript) after treatment but the transcripts in Nip showed no difference between control and treatment. “***” indicated that the *p*-value was less than 0.001.


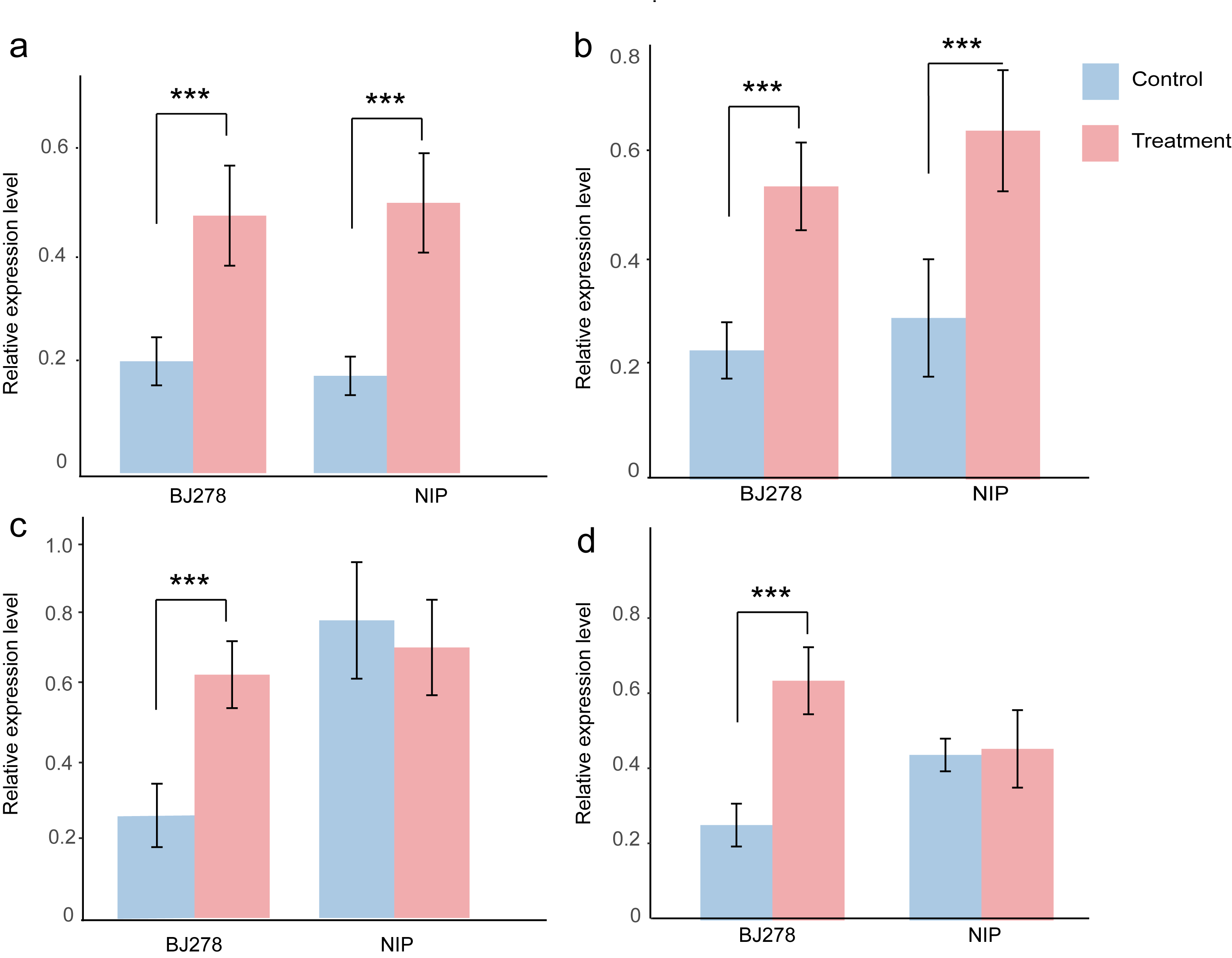


Figure S12. Venn diagram of coding–noncoding NAT pairs of which enriched in GO terms related to drought stress in Nip, BJ89, and BJ278.


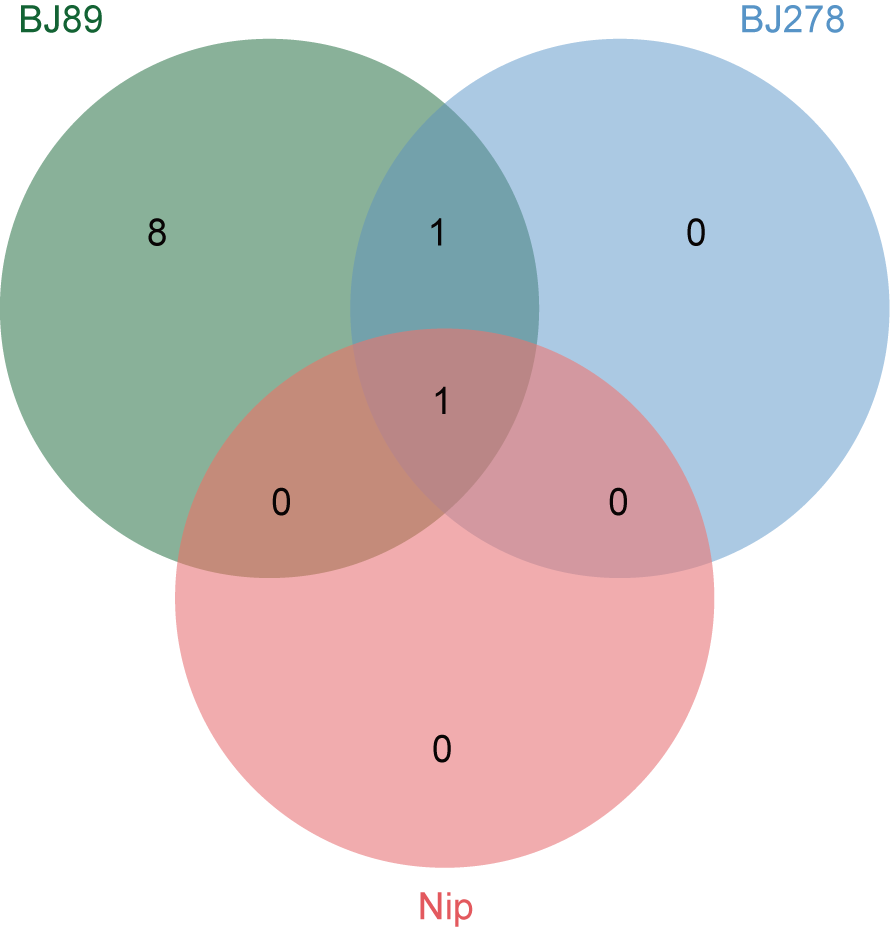


Figure S13. Sequence alignments of 10 coding–noncoding NAT pairs related to GO enrichment terms for the drought stress response. The top sequence was sense transcript and the sequence below was antisense transcript.

NAT pair for MSTRG.1295 and Os01t0256500-02


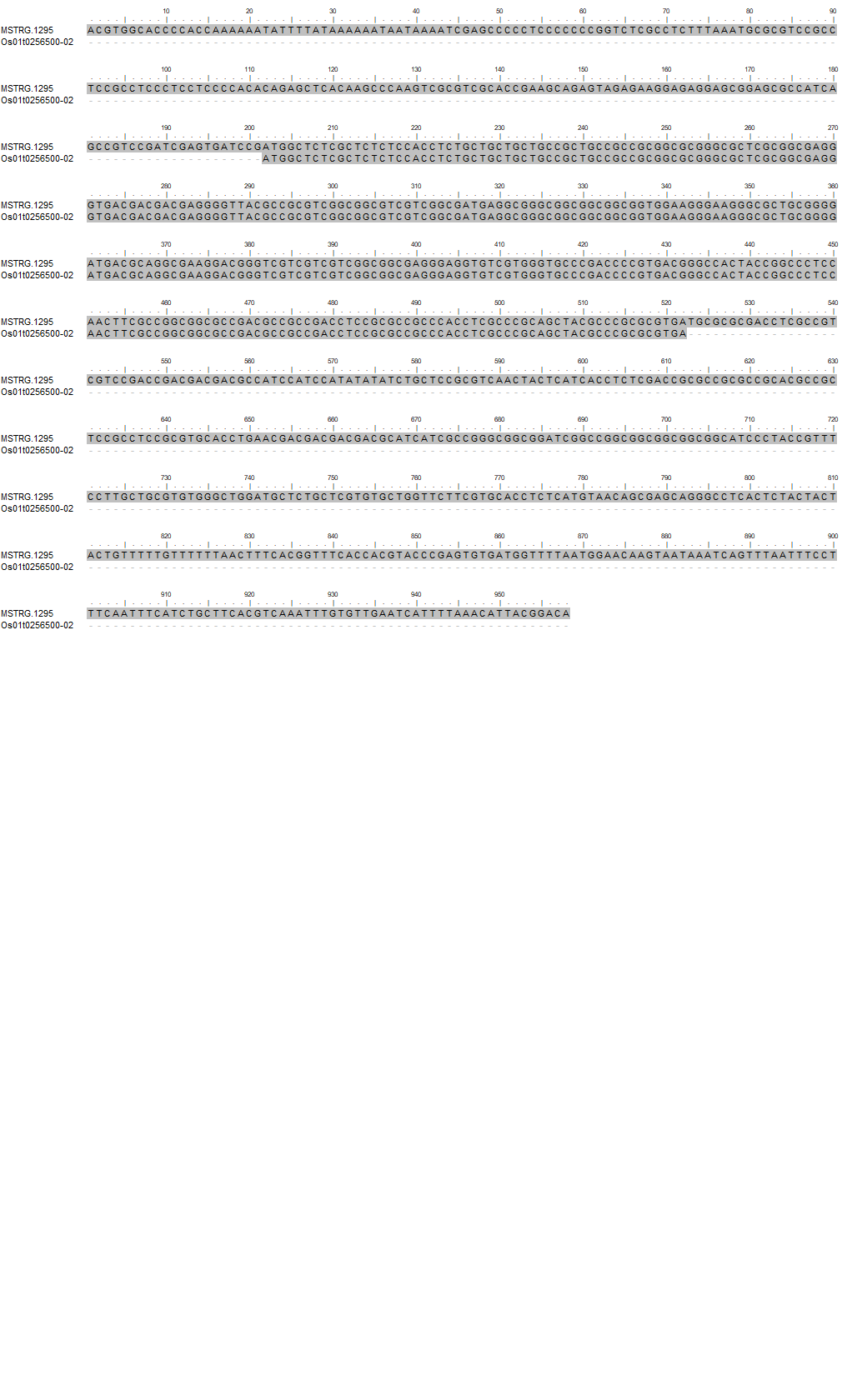


NAT pair for MSTRG.12385 and Os03t0402800-01


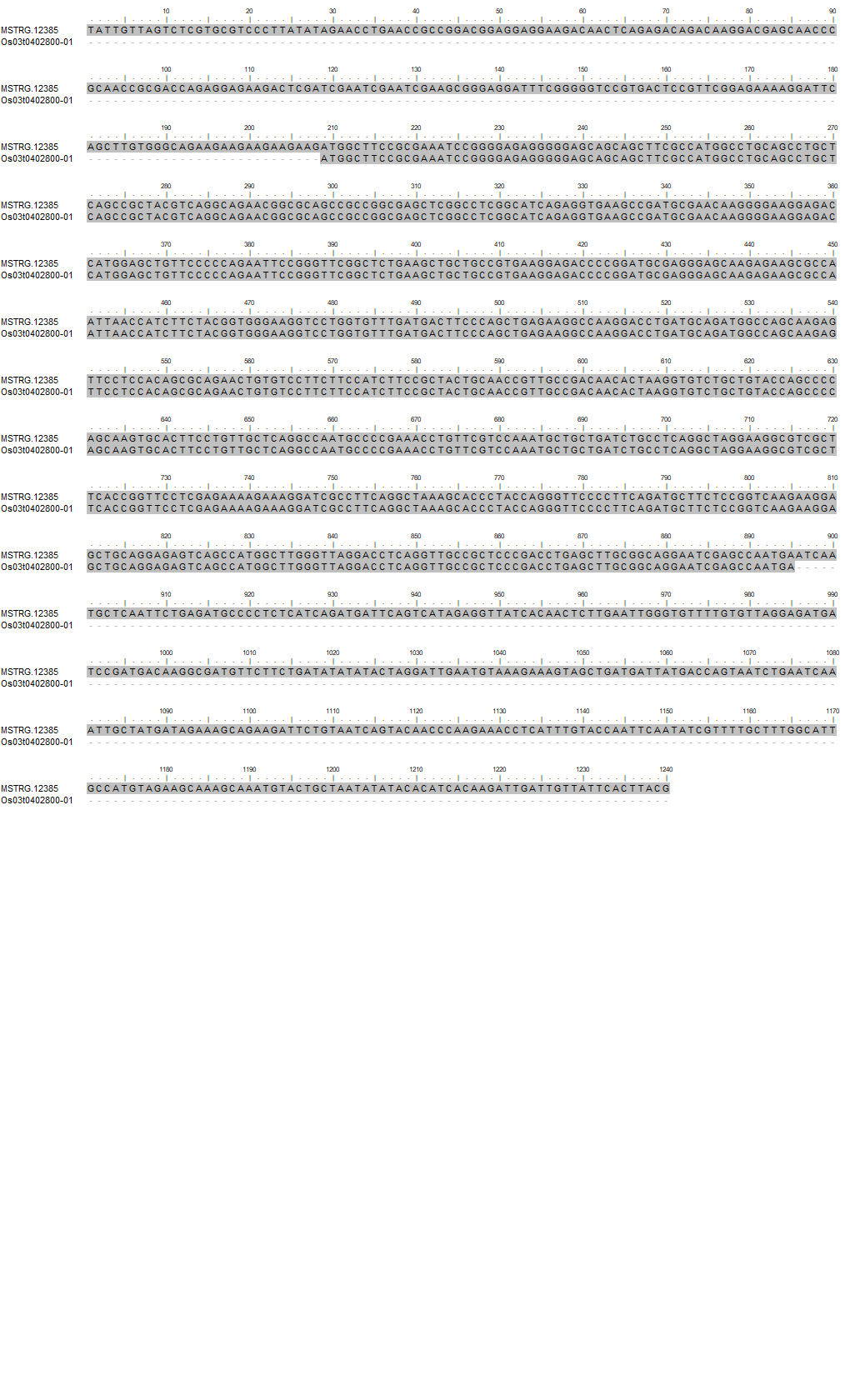


NAT pair for MSTRG.12385 and Os03t0402800-02


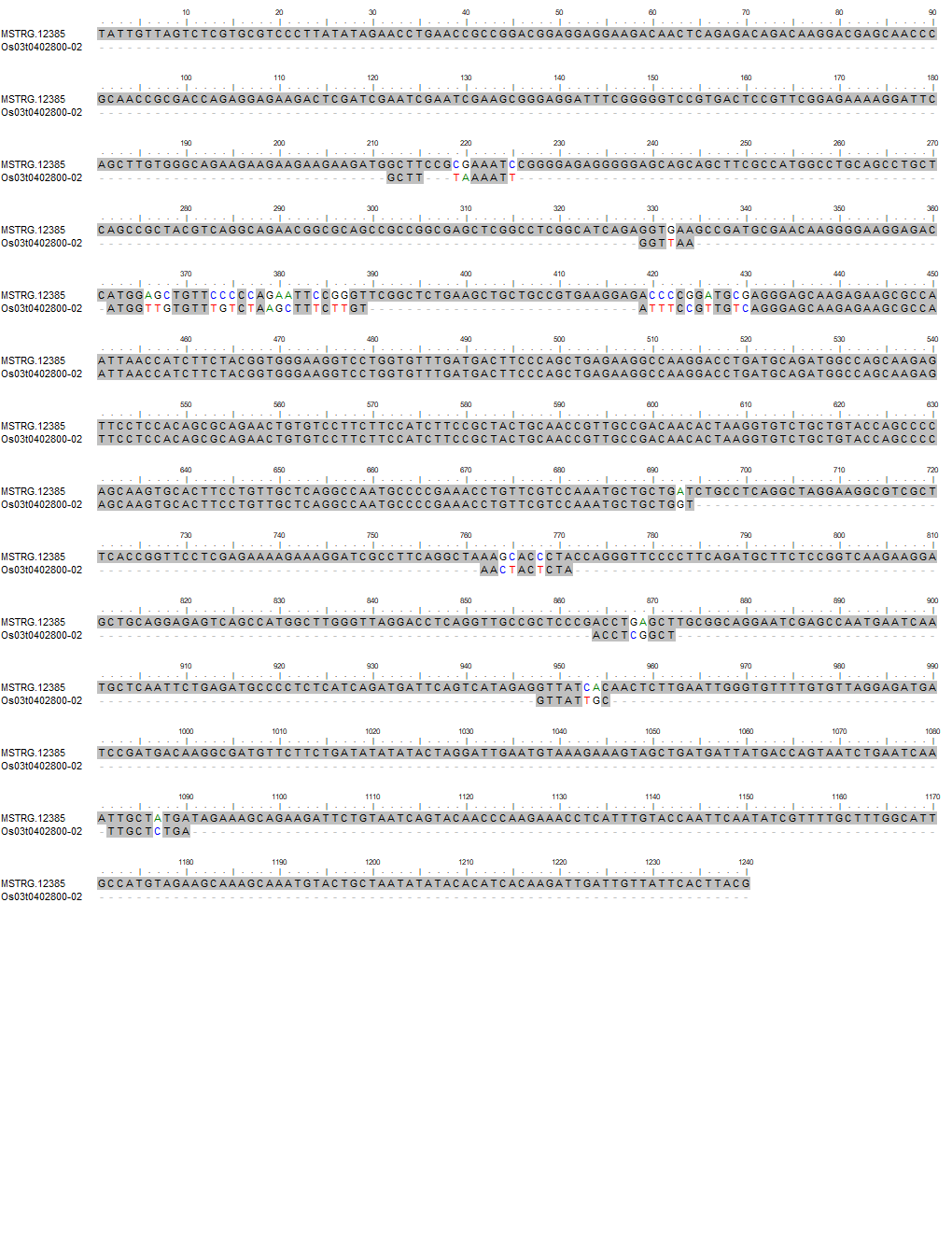


NAT pair for Os03t0161900-01 and MSTRG.10570


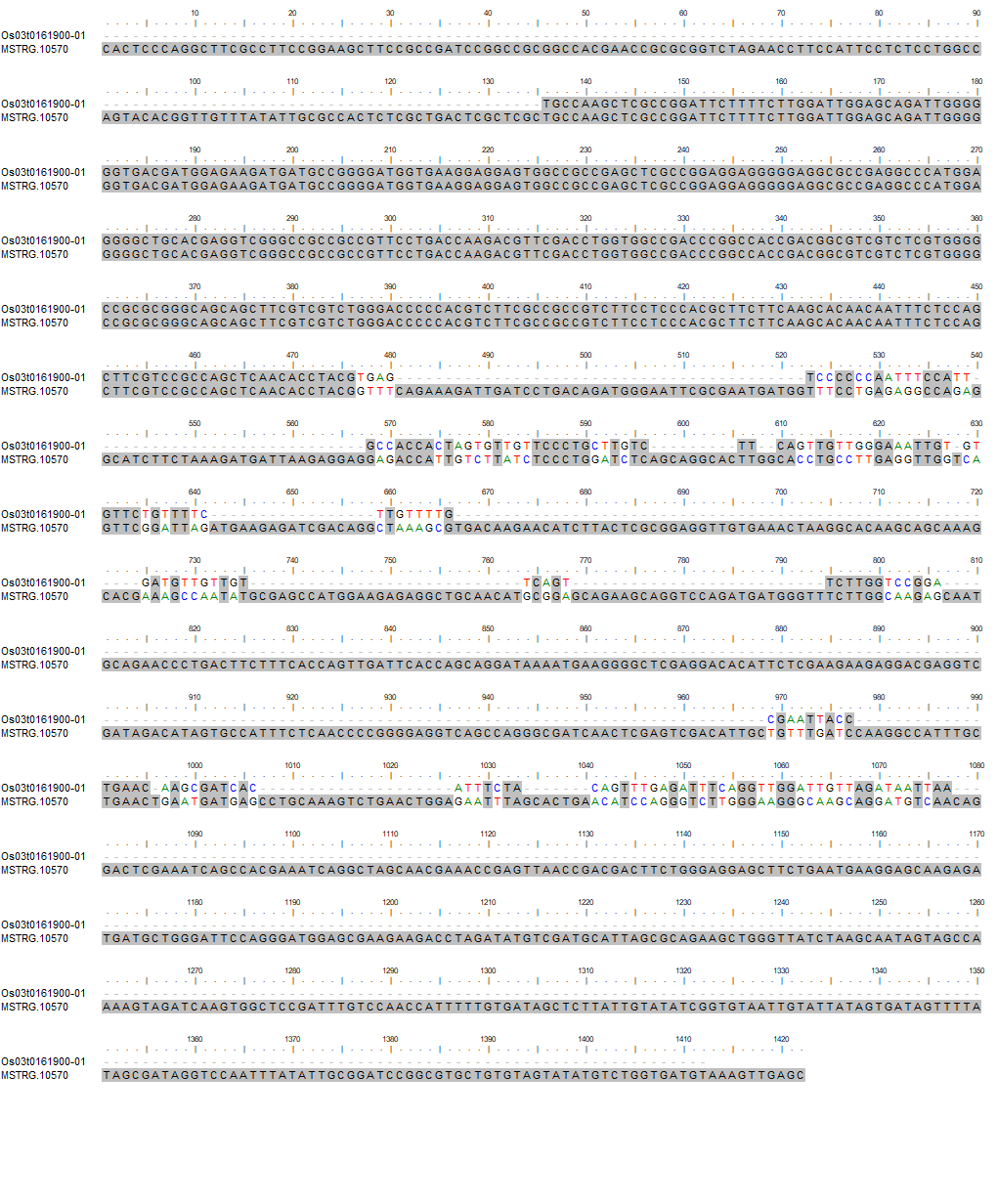


NAT pair for Os03t0161900-02 and MSTRG.10570


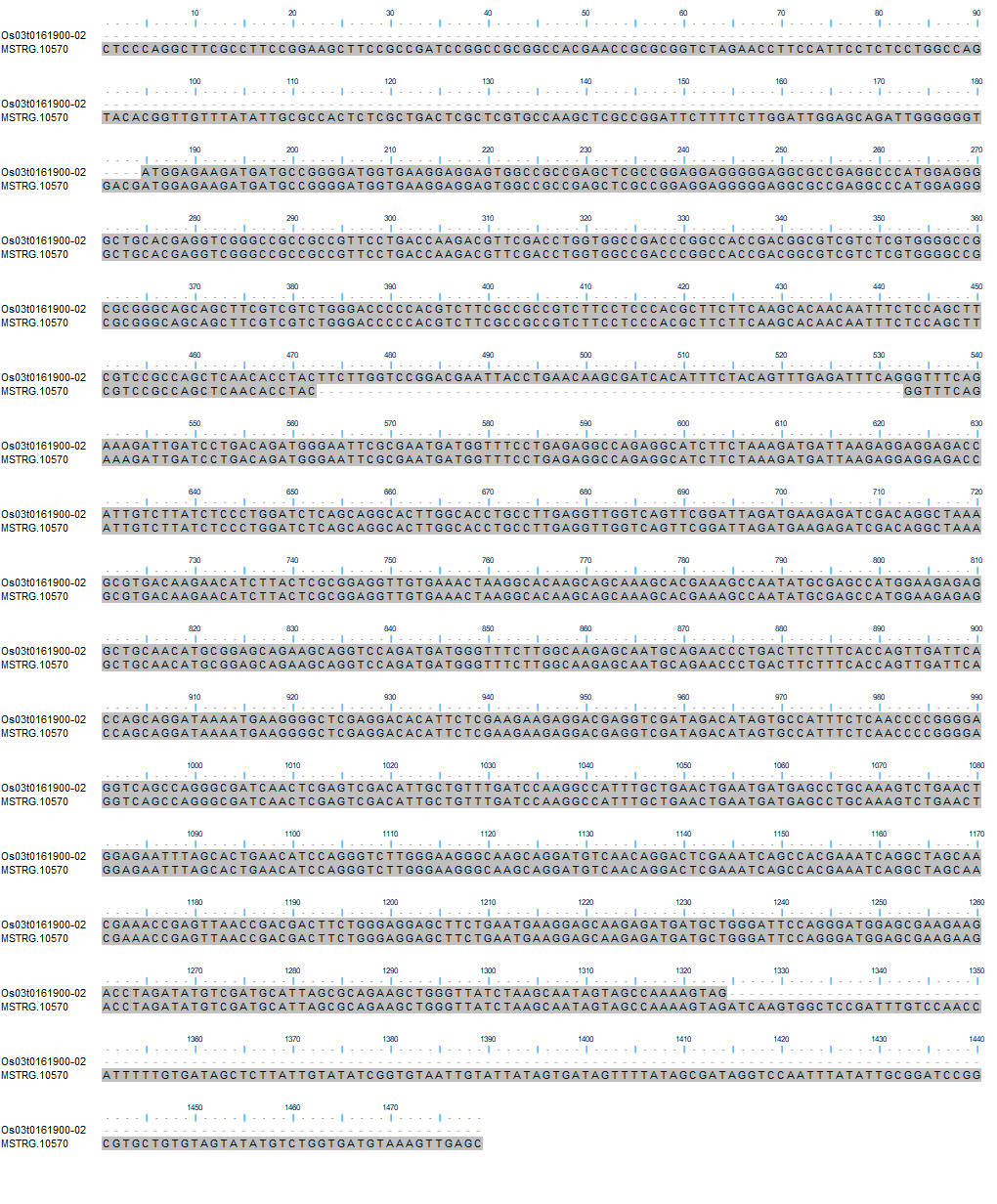


NAT pair for Os04t0497700-01 and MSTRG.16704


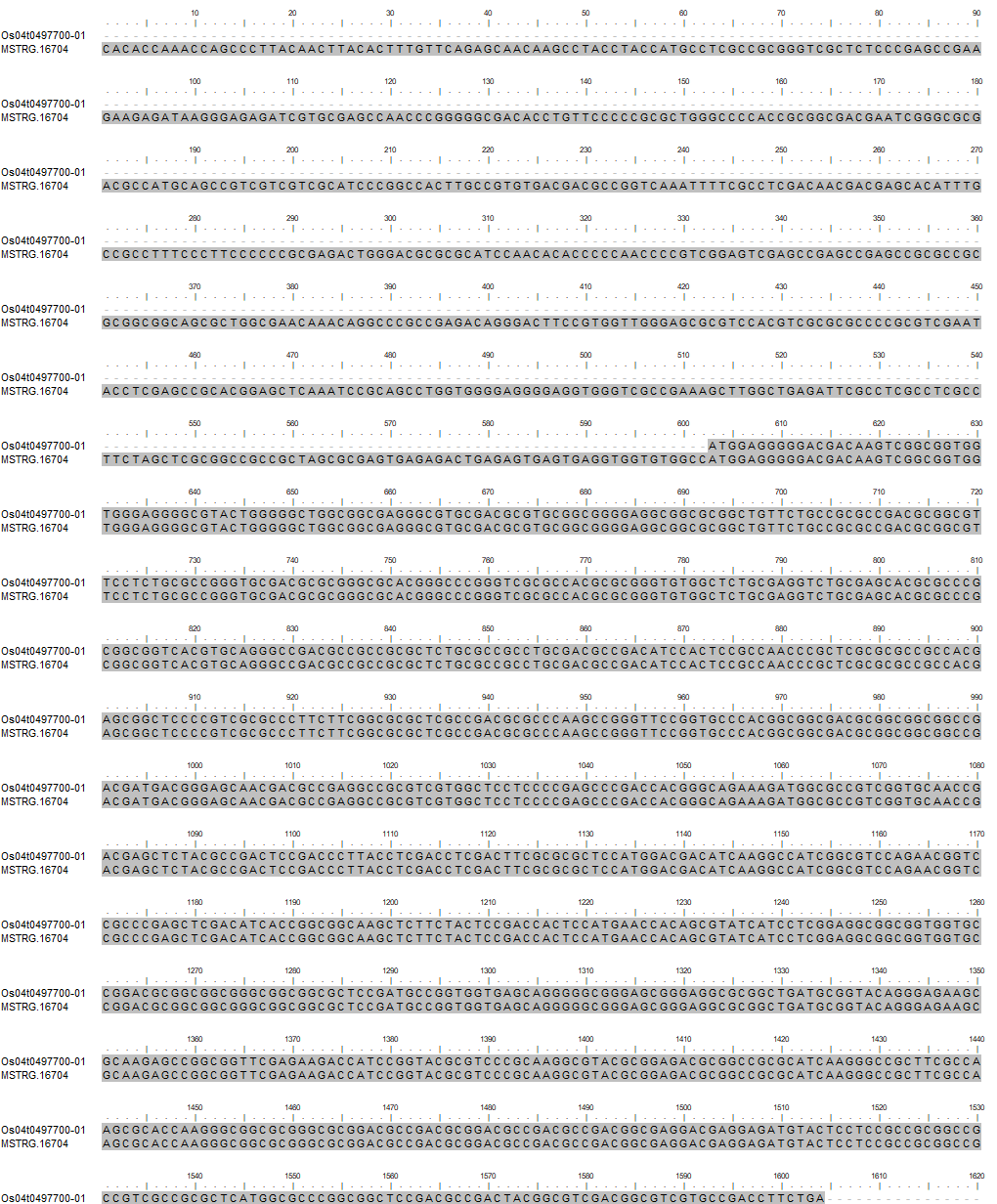


NAT pair for Os07t0615200-01 and MSTRG.27311


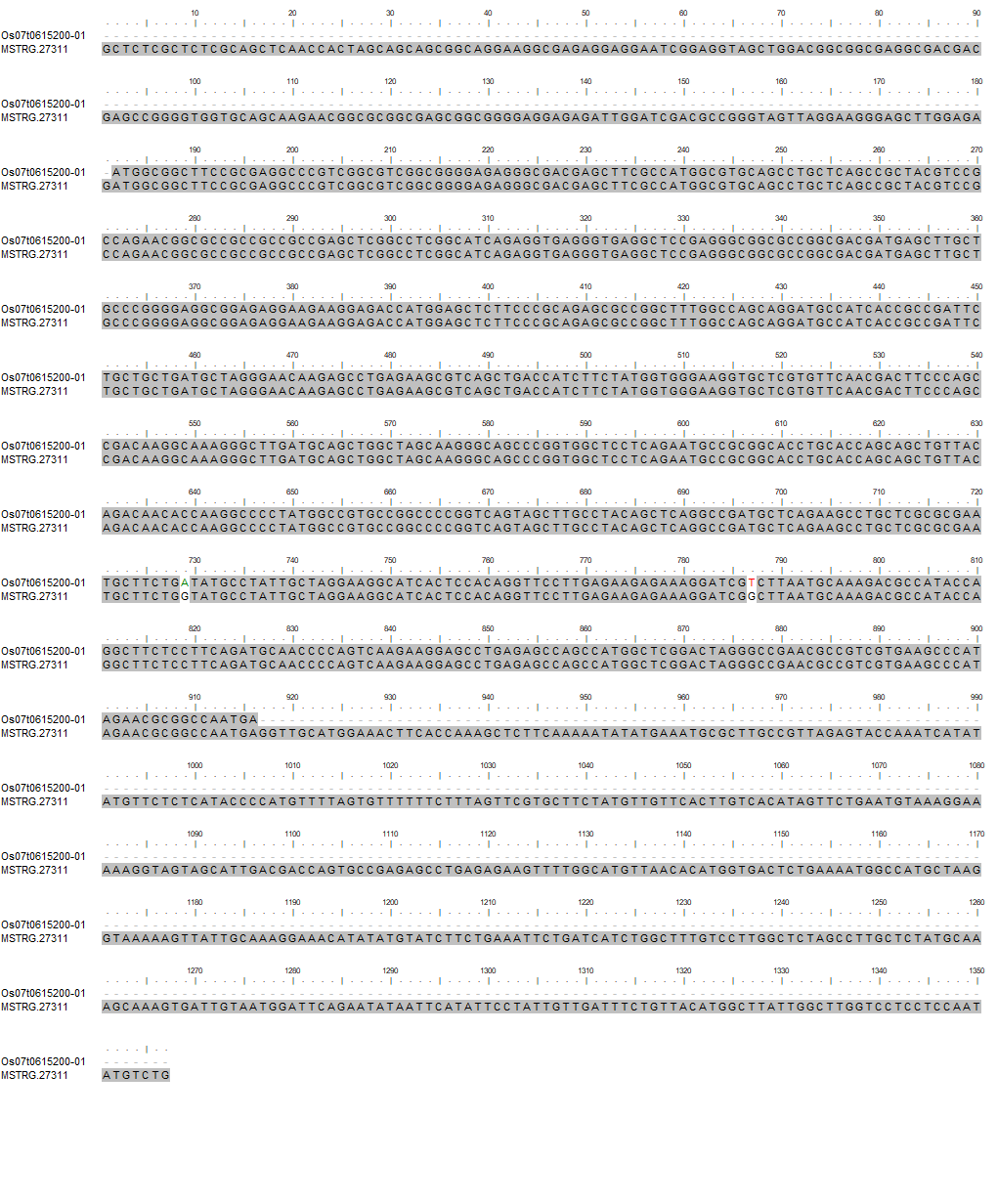


NAT pair for Os08t0504700-01 and MSTRG.30183


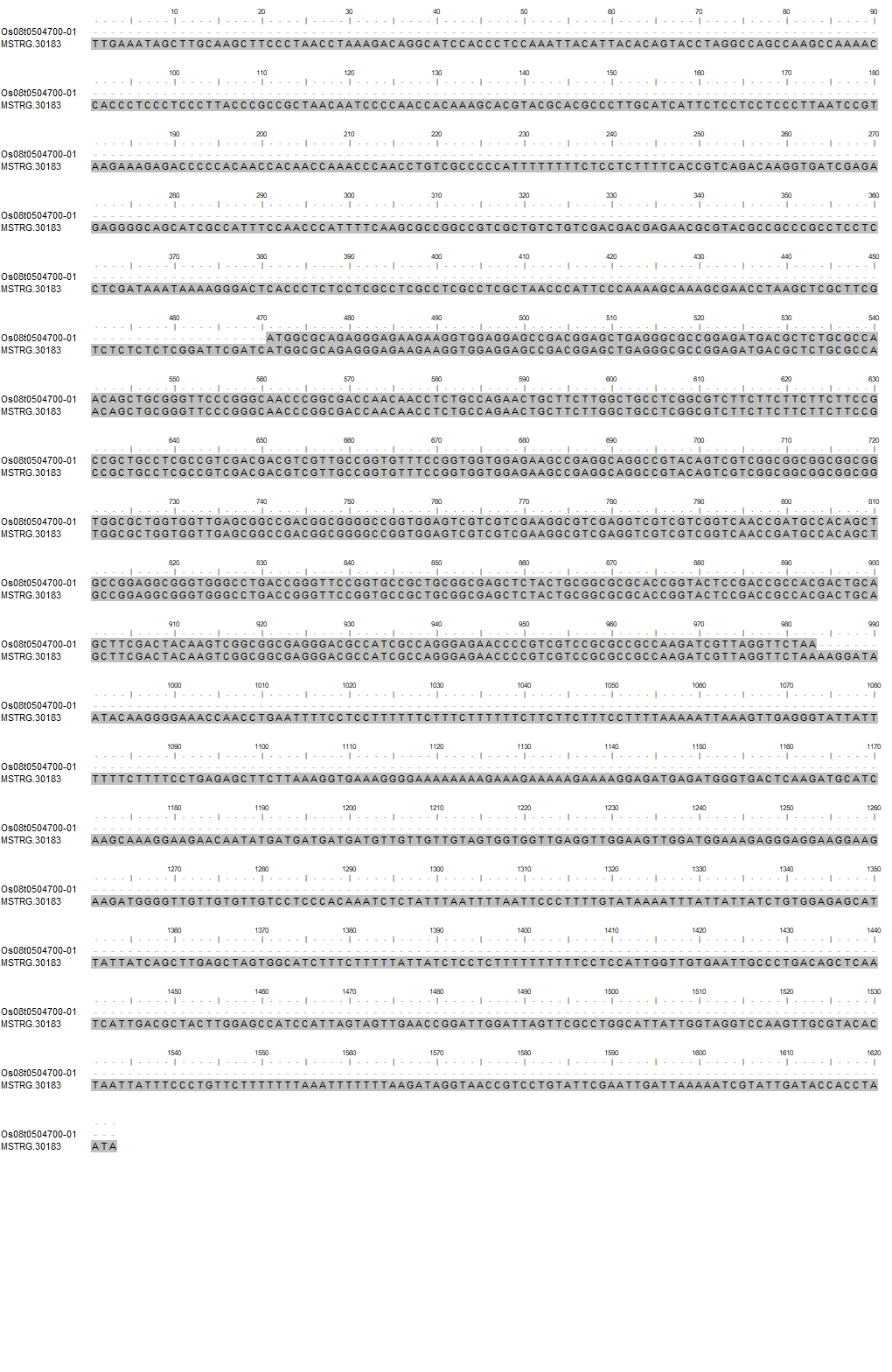


NAT pair for MSTRG.30767 and Os09t0106700-01


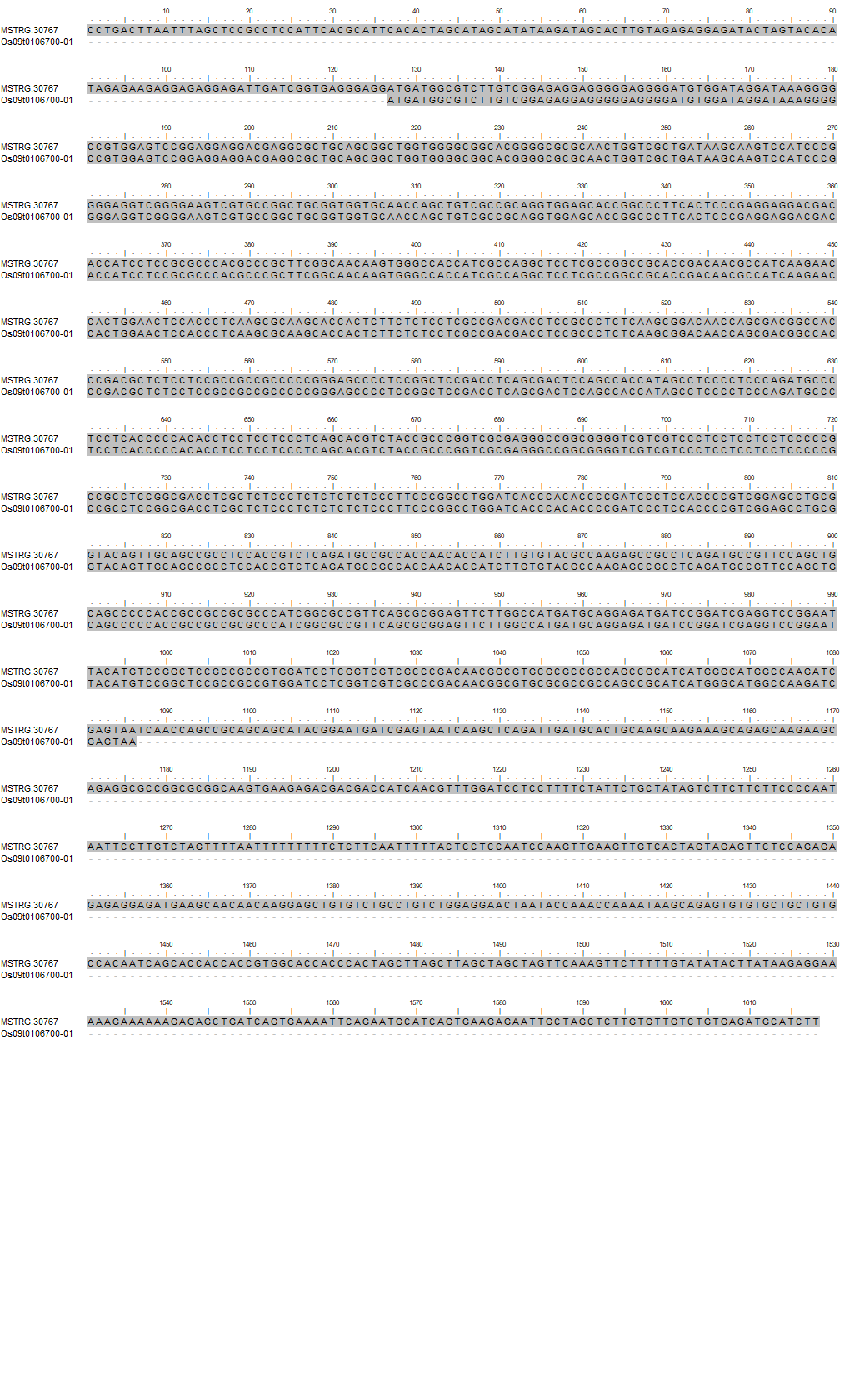


NAT pair for MSTRG.31075 and Os09t0273600-00


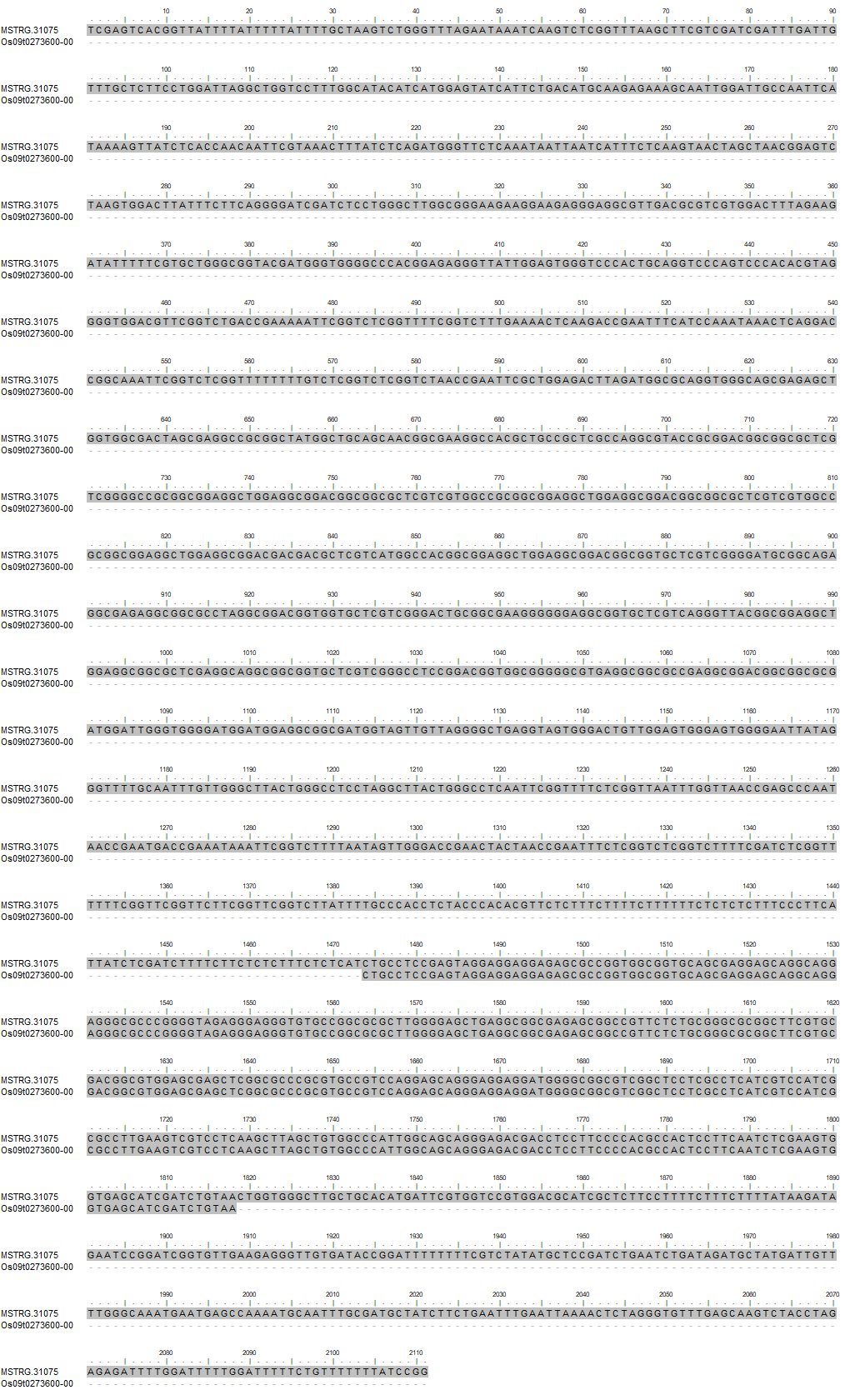


Table S1. Statistics of paired-end reads generated by ssRNA-seq analysis of 18 cDNA libraries of Nipponbare (Nip), BJ89, and BJ278 samples treated with or without drought stress.

| Sample | Clean reads （pair end） | Perfect match |
| --- | --- | --- |
| BJ278C1 | 26,550,050 | 21,889,911 |
| BJ278C2 | 27,483,610 | 22,035,970 |
| BJ278C3 | 22,154,962 | 18,051,372 |
| BJ278P1 | 21,691,338 | 17,040,510 |
| BJ278P2 | 22,115,769 | 18,627,593 |
| BJ278P3 | 22,688,076 | 19,762,471 |
| BJ89C1 | 22,969,595 | 20,078,905 |
| BJ89C2 | 23,224,610 | 19,787,140 |
| BJ89C3 | 24,622,291 | 21,218,229 |
| BJ89P1 | 27,849,875 | 23,251,262 |
| BJ89P2 | 29,600,090 | 24,763,579 |
| BJ89P3 | 30,706,772 | 26,070,880 |
| NC1 | 21,995,599 | 18,058,680 |
| NC2 | 20,166,961 | 15,714,860 |
| NC3 | 24,953,356 | 22,124,793 |
| NP1 | 23,683,666 | 19,754,476 |
| NP2 | 27,668,062 | 23,102,388 |
| NP3 | 25,383,309 | 22,311,393 |

Table S7. List of primers used in this study for quantitative PCR.

| Primer | Sequence(5' to 3') | Transcript | description |  |
| --- | --- | --- | --- | --- |
| primer1F | GTATGGAGGAGCCAAGGT | Os02t0258800-01 | sense transcript |  |
| primer1R | TTAGGTTACAAACTGTAGGTGC |  |  |  |
| primer2F | GCCAACAATCAAACACTAAA | MSTRG.6860.1 | antisense transcript |  |
| primer2R | GGTGGTGCTACTCGCTATG |  |  |  |
| primer3F | GCAAATGGTCGGACTTACAC | Os02t0504000-01 | sense transcript |  |
| primer3R | AATCCCAGAGGACTCACCC |  |  |  |
| primer4F | GAGGGAGAAGAGTGAGGA | MSTRG.7513.1 | antisense transcript | |
| primer4R | ATCTATACCGGACGAAAC |  |  |  |
| primer5F | TGGTCGTACCACAGGTATTGTGTT |  | *actin* |  |
| primer5R | AAGGTCGAGACGAAGGATAGCAT |  |  |  |
